# Transcription Profiling of *Bacillus subtilis* Cells Infected with AR9, a Giant Phage Encoding Two Multisubunit RNA Polymerases

**DOI:** 10.1128/mBio.02041-16

**Published:** 2017-02-14

**Authors:** Daria Lavysh, Maria Sokolova, Marina Slashcheva, Konrad U. Förstner, Konstantin Severinov

**Affiliations:** a Institute of Molecular Genetics, Russian Academy of Sciences, Moscow, Russia; b Institute of Antimicrobial Chemotherapy, Smolensk State Medical University, Smolensk, Russia; c Skolkovo Institute of Science and Technology, Skolkovo, Russia; d Peter the Great St.Petersburg Polytechnic University, Saint-Petersburg, Russia; e Core Unit Systems Medicine, University of Würzburg, Würzburg, Germany; National Cancer Institute

## Abstract

Bacteriophage AR9 is a recently sequenced jumbo phage that encodes two multisubunit RNA polymerases. Here we investigated the AR9 transcription strategy and the effect of AR9 infection on the transcription of its host, *Bacillus subtilis*. Analysis of whole-genome transcription revealed early, late, and continuously expressed AR9 genes. Alignment of sequences upstream of the 5′ ends of AR9 transcripts revealed consensus sequences that define early and late phage promoters. Continuously expressed AR9 genes have both early and late promoters in front of them. Early AR9 transcription is independent of protein synthesis and must be determined by virion RNA polymerase injected together with viral DNA. During infection, the overall amount of host mRNAs is significantly decreased. Analysis of relative amounts of host transcripts revealed notable differences in the levels of some mRNAs. The physiological significance of up- or downregulation of host genes for AR9 phage infection remains to be established. AR9 infection is significantly affected by rifampin, an inhibitor of host RNA polymerase transcription. The effect is likely caused by the antibiotic-induced killing of host cells, while phage genome transcription is solely performed by viral RNA polymerases.

## IMPORTANCE

Phages regulate the timing of the expression of their own genes to coordinate processes in the infected cell and maximize the release of viral progeny. Phages also alter the levels of host transcripts. Here we present the results of a temporal analysis of the host and viral transcriptomes of *Bacillus subtilis* infected with a giant phage, AR9. We identify viral promoters recognized by two virus-encoded RNA polymerases that are a unique feature of the phiKZ-related group of phages to which AR9 belongs. Our results set the stage for future analyses of highly unusual RNA polymerases encoded by AR9 and other phiKZ-related phages.

## INTRODUCTION

During bacteriophage infection, multiple concerted changes in host and virus gene expression levels are observed (reference 1 and references therein). Most phages regulate the timing of the expression of their own genes to coordinate different biochemical processes and to maximize the release of viral progeny. Usually, genes of double-stranded DNA (dsDNA) phages are divided into three temporal expression classes, early, middle, and late. Early phage genes are the most diverse; some encode proteins that allow the virus to evade host defenses or condition the cell for productive infection. Middle genes usually encode viral DNA replication and recombination proteins and enzymes involved in nucleotide biosynthesis. Products of late genes are involved in virion formation, DNA packaging, and host lysis. Control of viral gene expression is most commonly exerted at the level of transcription and is organized in a cascade-like way to ensure the temporally ordered expression of genes belonging to different groups. Proteins that are required for the transcription of early genes are either host proteins or phage late gene products packaged inside virions and injected into infected cells along with viral DNA. Transcription factors required for middle gene expression are often encoded by early genes, while factors required for late transcription are usually the products of middle genes.

Many phages also alter the levels of host transcripts. Lytic phages usually decrease host transcription (2). For example, during *Escherichia coli* phage T4 infection, global destabilization of host mRNAs occurs (3). A dramatic reduction in the overall amounts of bacterial transcripts is also observed during infection with *Pseudomonas* phages PAK_P3 and LUZ19, *Lactococcus lactis* phage c2, and *Synechococcus* phage Syn9 (4–7). In some cases, levels of specific host transcripts are changed to ensure successful infection. An operon involved in RNA processing is upregulated during PAK_P3 infection (4). The products of two host genes that play an important role in amino acid metabolism are highly induced during LUZ19 infection (5). During phage c2 infection, genes whose products are involved in cell envelope synthesis, carbohydrate metabolism, and transcription regulation are differentially expressed (6). A carboxysome carbon fixation gene and a small heat shock-like chaperone gene are upregulated during Syn9 infection (7).

Most phages rely on a multisubunit RNA polymerase (RNAP) of a bacterial host to transcribe their genes. Transcription regulation during phage infection is carried out by phage-encoded proteins that bind to host RNAP. Some phages encode a sigma (promoter specificity) factor that binds to a host RNAP core enzyme and directs it to viral promoters of a particular class. Examples of this strategy include gp55 of phage T4 (8) and gp36 of phage phiEco32 (9). Another strategy is the use of phage-encoded proteins that modify the promoter specificity of the host RNAP holoenzyme. Well-studied examples include the bacteriophage T4 Alt, AsiA, and MotA proteins. The Alt protein modifies Arg^265^ on one of the two α subunits of RNAP, enhancing transcription from some early promoters and decreasing transcription from host promoters (10, 11). AsiA and MotA bind to the host RNAP σ^70^ holoenzyme and redirect it from early to middle phage promoters. Other examples include the *E. coli* phage N4 single-stranded binding (SSB) protein (redirects the σ^70^ holoenzyme from host to late viral promoters [12]), the *Thermus* phage P23-45 gp39 and gp76 proteins, and the *Xanthomonas oryzae* Xp10 phage protein p7 (switches off host gene transcription but is required for late gene transcription by the host RNAP holoenzyme [13]).

Yet another strategy used by some phages is to rely on phage-encoded single-subunit RNAPs for the transcription of specific groups of phage genes. A classic example is bacteriophage T7, which encodes a single-subunit RNAP, a product of an early phage gene that transcribes the middle and late genes of the phage from specific promoters (14). The switch from host RNAP transcription of bacterial and early phage promoters to T7 RNAP transcription of middle and late promoters is accomplished by host RNAP inhibitor gp2, a product of an early gene (15, 16). Bacteriophage N4 presents another example. It encodes two RNAPs. One is injected into infected cells together with viral DNA and transcribes early phage genes (17). The second RNAP transcribes middle phage genes (18). Late genes of the phage are transcribed by host RNAP and require a viral SSB protein (see above).

An even more radical strategy is used by a giant *Pseudomonas aeruginosa* phage, phiKZ. Its development is independent of host RNAP transcription but relies on two phage-encoded multisubunit RNAPs, one carried by a virion and another produced in infected cells (19). Transcriptomic analysis of phiKZ infection has been carried out, and three classes of viral promoters were revealed. Early transcription must be carried out by virion RNAP (vRNAP) and does not require viral protein synthesis. The late and possibly middle promoters are recognized by non-vRNAP (nvRNAP), a product of early genes (20). The nvRNAP transcribes, among others, vRNAP subunit genes. The vRNAP produced late in infection must be kept inactive to prevent out-of-time early gene transcription before it is packaged into progeny virions.

PhiKZ is a founding member of a highly diverse group of phiKZ-related phages whose characteristic feature is the presence of two sets of RNAP subunit genes, of which one resides in the virion. A recently recognized member of the phiKZ-related phage group is *Bacillus subtilis* phage AR9 (21), a pseudolysogenic phage capable of general transduction (22). Along with its close relatives PBS1/2, AR9 has been widely used to construct early genetic maps of the *B. subtilis* genome (23). While it was reported that phiKZ could also establish a pseudolysogenic state by an unknown mechanism (24), most of the known members of the phiKZ family are considered lytic. In this work, we performed global transcription profiling to identify temporal expression classes of AR9 genes and define phage promoters. The results lay the foundation for future analyses of highly unusual RNAPs encoded by AR9 and other phiKZ-related phages.

## RESULTS

### One-step growth of the AR9 phage.

**FIG 1:**
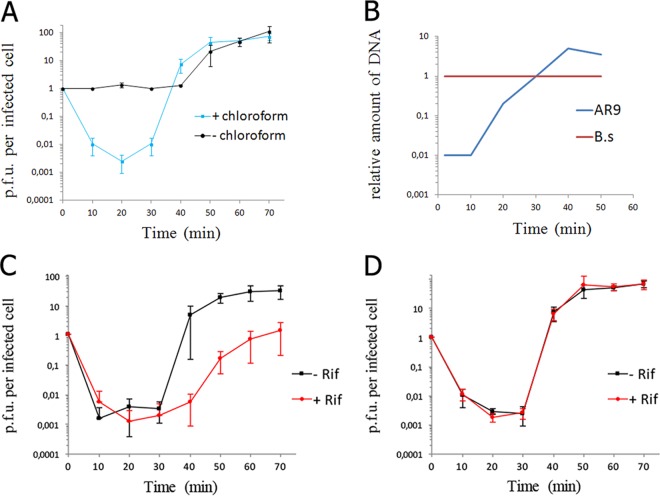
General properties of the AR9 infection process. (A) AR9 one-step growth curve on *B. subtilis* 168. The numbers of PFU per infected cell in chloroform-treated and untreated cultures are shown. (B) Accumulation of phage AR9 DNA during infection determined by qPCR analysis of DNA extracted at different time points after phage infection. The results obtained for the AR9 gene *g283* and the *B. subtilis* (B.s) gene *yoxA* in three independent reactions are shown. (C) AR9 one-step growth curve in the presence of the host RNAP inhibitor rifampin (Rif). The culture was chloroform treated prior to PFU determination. (D) AR9 one-step growth curve on a *B. subtilis* 168 mutant resistant to rifampin in the presence of rifampin. The culture was chloroform treated. For every one-step growth curve, each data point is an average of three biological replicates; error bars indicate standard deviations.

Infection of *B. subtilis* by the AR9 phage was analyzed in LB medium at 37°C. The latency period was found to be ca. 40 min, and phage progeny release was complete by 50 min postinfection (Fig. 1A). The burst size was estimated at ~80 PFU per infected cell. The amount of phage DNA in infected cells started to increase at about 20 min postinfection and reached a maximum at 40 min postinfection (Fig. 1B). Infections were also repeated in the presence of rifampin, an inhibitor of transcription by host RNAP. Although a reproducible increase in the phage titer at the end of the infection was observed in the presence of rifampin, indicating clear phage progeny formation, the yield of the phage was decreased ~10-fold compared to that in a control infection (Fig. 1C). No phage progeny production is detected during the infection of rifampin-treated cells with phages that rely on host RNAP for the expression of at least one temporal class of their genes. Therefore, it appears that AR9 infection can proceed in the absence of host RNAP transcription.

The decrease in AR9 progeny production in the presence of rifampin was specific, since no inhibition of viral development was observed when host cells carrying a spontaneous rifampin resistance *rpoB* mutation substituting RNAP β subunit Ser487 for Leu were infected in the presence of rifampin (Fig. 1D). The results appeared to contradict earlier data showing that the development of PBS2, a phage that is highly related to AR9 (21), was insensitive to rifampin (25). The difference is likely due to greater killing of the *B. subtilis* 168 bacterium used as a host for AR9 infection by rifampin than of the PBS2 infection host. Indeed, treating an uninfected *B. subtilis* 168 culture with rifampin for 10 min decreased the number of CFU by 2 orders of magnitude (see Fig. S1 in the supplemental material), while treating another laboratory *B. subtilis* strain with rifampin had both a less detrimental effect on cell survival and a smaller inhibitory effect on phage yield (Fig. S2). Given these observations, it appears that AR9 infection can proceed in the absence of transcription by host RNAP.

10.1128/mBio.02041-16.1 FIG S1  *B. subtilis* 168 growth with/without the addition of rifampin. Numbers of CFU in *B. subtilis* cultures (OD_595_ of 0.5, 0 min on the bar graph) in LB medium (gray rectangles) and in LB medium with rifampin added to a final concentration of 50 μg/ml (red rectangles) are shown. Download FIG S1, TIF file, 0.02 MB. Copyright © 2017 Lavysh et al. 2017 Lavysh et al. This content is distributed under the terms of the Creative Commons Attribution 4.0 International license.

10.1128/mBio.02041-16.2 FIG S2  Increased resistance of the host to killing by rifampin leads to increased production of AR9 progeny in the presence of rifampin. (A) Numbers of CFU of *B. subtilis* 168 and *B. subtilis* laboratory strain cultures (OD_595_ of 0.5) formed after 10 min of incubation with rifampin are shown (red rectangles). Gray rectangles show the results of control experiment (no rifampin added). (B) AR9 one-step growth curve on the *B. subtilis* 168 host in the presence or absence of rifampin. The number of PFU per infected cell is shown. (C) AR9 one-step growth curve on a laboratory strain of *B. subtilis* in the presence or absence of rifampin. Download FIG S2, TIF file, 0.1 MB. Copyright © 2017 Lavysh et al. 2017 Lavysh et al. This content is distributed under the terms of the Creative Commons Attribution 4.0 International license.

### Transcription profiling of AR9.

**FIG 2:**
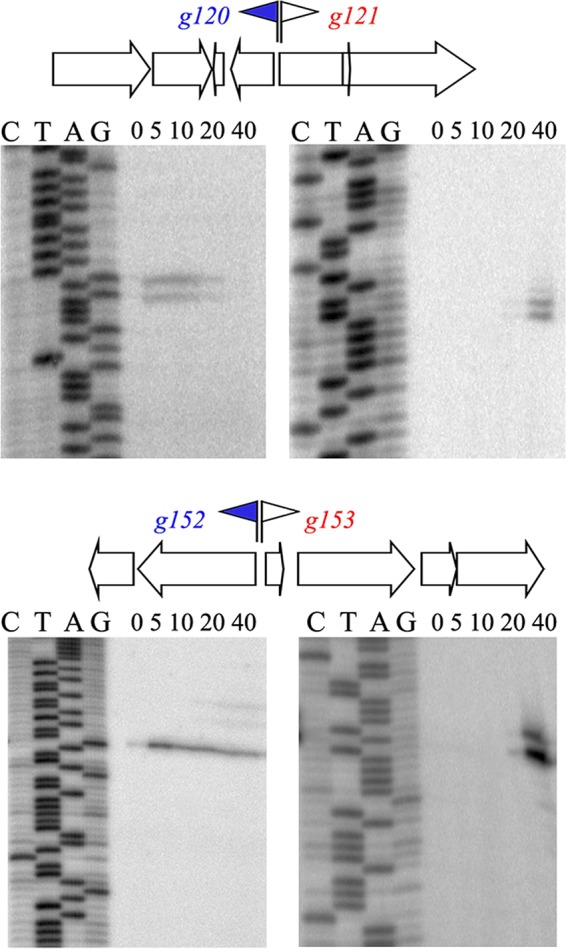
Temporal patterns of expression of selected AR9 genes. Primer extension reactions were conducted with RNA prepared from AR9-infected cells with primers annealing to regions separating the oppositely transcribed AR9 genes indicated. Time postinfection (in minutes) is indicated at the top of each gel. Early promoters and corresponding genes are blue, and late genes are red.

To establish characteristic times of accumulation of AR9 transcripts from different expression classes, we performed primer extension reactions by using as a template RNA purified from infected cells (Fig. 2). We used primers that annealed around two intragenic AR9 regions separating oppositely oriented genes. The regions chosen for analysis contain predicted putative early promoters in front of genes *g120* and *g152* and should contain oppositely oriented non-early promoters upstream of virion protein genes *g121* and *g153*. Indeed, primer extension products corresponding to *g120* and *g152* transcripts were present 5 min postinfection and persisted at later times (30 and 40 min). In contrast, transcripts of *g121* and *g153* appeared only by 20 min of infection.

On the basis of the one-step growth curve and primer extension experiment results, we selected three time points of infection (5, 20, and 40 min) to perform global profiling of both AR9 and host transcripts. We expected to detect early phage transcripts at 5 min of infection; middle or late transcripts were expected to start appearing at 20 min postinfection. The 40-min time point was chosen to see whether early and middle transcription is shut off at a time close to the end of the infection cycle. Since 20-min postinfection transcripts of different temporal classes are simultaneously detected in infected cells, we also performed differential RNA sequencing (dRNA-Seq) analysis of RNA collected at this time point to identify 5′ ends of primary phage transcripts. Overall, 73 to 83% of all reads were aligned with the *B. subtilis* and AR9 genomes in a strand-specific manner. The number of reads that corresponded to the AR9 genome progressively increased from 10% for the 5-min library to 21% for the 20-min library, and finally to 52% for the 40-min library.

During dRNA-Seq, one portion of the total RNA was treated with a 5′ monophosphate-dependent terminator exonuclease (TEX), which degrades 5′ monophosphate RNA, resulting in enrichment of RNA molecules containing 5′ triphosphates compared to untreated RNA. TEX treatment resulted in robust enrichment of signals from known host promoters, validating the procedure (data available on request). Compared to host primary transcripts, the AR9 transcript ends were much less responsive to TEX treatment. Only 61 AR9 RNA 5′ ends were significantly enriched in the +TEX library. These included 18 transcripts preceded by a predicted early promoter consensus motif (21). Since there are 34 predicted early promoters on the basis of the occurrence of an overrepresented consensus motif (21), to retrieve additional early transcripts, the threshold for “enrichment factor” of the TSSPredator program used to predict 5′ ends from dRNA-Seq data was decreased to 0.1 from the 2.0 default setting. In this way, 21 additional 5′ ends associated with the early consensus motif whose abundance was marginally decreased after TEX treatment were identified. For five 5′ ends associated with the predicted early consensus motif, TEX treatment led to a significant (>2-fold) decrease in abundance (Table S2). Despite the differences in responses to TEX treatment, we consider all of these 5′ ends *bona fide* primary transcripts because in each case, in addition to the presence of the putative early promoter consensus motif, there was a clear enrichment of 5′-proximal reads compared to reads more downstream, which is a characteristic behavior of RNA originating from promoters (26). Analysis of the RNA-Seq time series data shows that all of the primary transcripts from this group appear early in infection (see below). The lack of enrichment for early phage transcripts upon TEX treatment may be caused by dephosphorylation of primary transcripts by 20 min of infection, a time point used for dRNA-Seq library preparation.

One hundred ten AR9 RNA 5′ ends that lacked the early consensus motif in front of them were found with the relaxed TSSPredator setting and were also considered transcriptional start sites (TSSs) (Table S3). Eight additional TSSs were included after manual curation of the 40-min RNA-Seq library. For these transcripts, no 5′ ends were found at the 20-min time point (NA in all lines in Table S3), but clear accumulation at the later point was observed.

**FIG 3:**
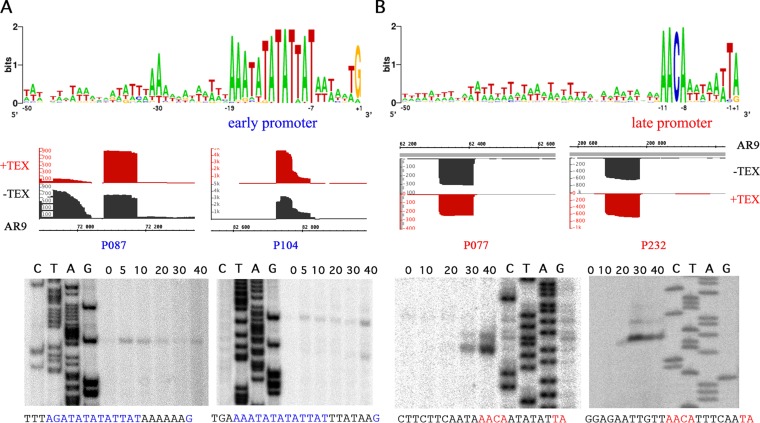
Early and late promoters of bacteriophage AR9 revealed by dRNA-Seq analysis of phage transcripts. Consensus sequences for early (A) and late (B) promoters are shown at the top. The dRNA-Seq data for two early (P087 and P104) and two late (P077 and P232) promoters are presented below. Regions at the 5′ ends of selected AR9 genes from cDNA libraries without (black) or with (red) TEX treatment are presented (the AR9 genome coordinates are shown at the bottom [A] and top [B], since corresponding early and late genes are transcribed from different strands of the genome). At the bottom, primer extension data validating the dRNA-Seq results along with the corresponding sequencing reactions are shown. Time postinfection (in minutes) is shown at the top of each gel. Below each primer extension gel, the sequence upstream of the primer extension endpoint is shown, with consensus elements highlighted in colors.

Randomly chosen transcripts with or without the preceding predicted early promoter consensus sequence and with or without signal enrichment in +TEX libraries were tested by primer extension. In each case, the presence of the expected primer extension product was observed (Fig. 3).

Following the procedure described above, a total of 159 AR9 TSSs were identified upstream of 134 out of 293 previously annotated AR9 genes (292 open reading frames [ORFs] and 1 tRNA gene). Some genes had two TSSs in front of them. Alignment of sequences from −50 to +1 relative to the identified TSS clearly separates all of the sequences into two classes. Forty-one TSSs are associated with the early consensus motif AAATATATATTAT-(6N)-G (Fig. 3A). They include 33 out of the 34 early promoters identified bioinformatically on the basis of motif overrepresentation (21). A predicted early promoter in front of *g057* was not detected by dRNA-Seq. An early promoter in front of *g044* was not identified earlier because it deviates from the consensus sequence at the transcription start point, and another early promoter before gene *g190* was considered putative in our previous publication (21). The present analysis shows that *g190* is indeed an early gene with its own promoter. Both the gene and the promoter are located within an intron located in late gene *g189*. Regions upstream of genes *g083*, *g159*, and *g226* contain correctly predicted early promoters, but each also harbors an additional early promoter whose consensus element is shifted with respect to the predicted ones and was missed during the earlier analysis.

TSSs for non-early phage transcripts were identified in front of 118 AR9 genes. Of these TSSs, 94 are associated with a consensus motif, AACA-(6N)-TA/G, where the last A or G is the TSS (Fig. 3B; Table S3). We consider this motif an AR9 late promoter consensus element. Seventeen late promoters either have different distances between the conserved AACA tetranucleotide and the TA/G element or carry one or two nonconsensus bases. Seven late promoters do not share similarities with the consensus motif. As mentioned above, eight late promoters are active only at the 40-min time point. Thirteen late promoters are internal, i.e., are located inside phage ORFs, and seven drive antisense transcription. Internal promoters can generate RNA variants from which N-terminally truncated copies of viral proteins may be expressed. The antisense RNAs are the most abundant for the genes *g125* and *g126* but are also detected for the genes *g002*, *g076*, *g090*, *g142*, *g167*, *g177*, *g194*, *g233*, *g240*, *g242*, and *g251*. Most antisense transcripts are detected at the end of the infection cycle. A notable exception is the antisense transcript of gene *g233*, which is only detectable at the 20-min time point. The sequences and other characteristics of all of the promoters are available in Tables S2 and S3.

Mapping of TSSs allowed us to identify the 5′ untranslated regions (UTRs) of the AR9 genes listed in Tables S2 and S3. The lengths of phage UTRs vary between 26 and 491 nucleotides (nt), with no significant differences between early and late gene transcripts. Long UTRs are unusual for phage transcripts, which are often leaderless (27). Some of the longer AR9 5′ UTRs may control the translational efficiency of downstream genes. Indeed, two motifs, one within the 5′ UTRs of early transcripts and another within those of late transcripts, were predicted (Fig. S3). The functional significance of these motifs remains to be established.

10.1128/mBio.02041-16.3 FIG S3  Putative motifs of long 5′ UTRs of AR9 mRNAs. (A) A motif found in early mRNAs *g214*, *g226*, *g248*, and *g270*. (B) A motif found in late mRNAs *g103*, *g125*, *g134*, *g158*, and *g261*. Download FIG S3, TIF file, 0.1 MB. Copyright © 2017 Lavysh et al. 2017 Lavysh et al. This content is distributed under the terms of the Creative Commons Attribution 4.0 International license.

### Temporal kinetic classes of AR9 transcripts.

**FIG 4:**
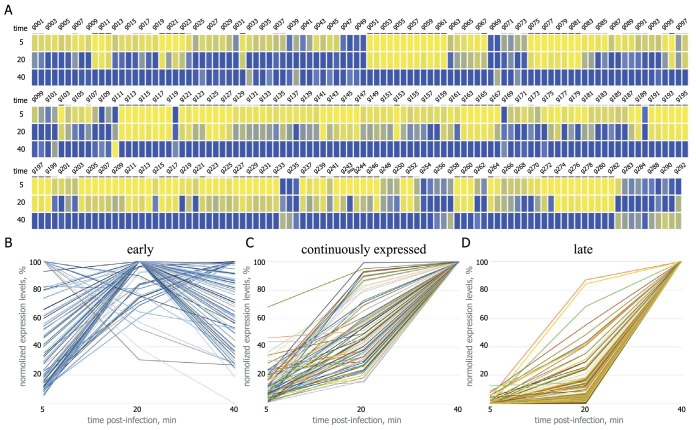
Global temporal patterns of AR9 genome transcription. (A) A heat map summarizing the temporal pattern of AR9 transcripts. The normalized expression levels of each AR9 gene across three time points (5, 20, and 40 min postinfection) are color coded blue for high and yellow for low expression levels. Late genes are underlined in dark gray, and continuously expressed genes are underlined in light gray. (B to D) Time courses of accumulation of individual AR9 transcripts (292 ORFs and one tRNA gene) divided into three classes are shown. The *y* axes show normalized gene expression levels.

To monitor transcript accumulation over the course of infection and estimate transcript abundances, we calculated the number of FPKM (fragments per kilobase of transcript per million mapped reads) for each AR9 gene (Table S4; Fig. 4A). The AR9 genes were classified on the basis of (i) when their transcripts were detected first, (ii) when their transcripts reached the highest abundance, and (iii) what class of promoter(s) is located upstream of the gene.

Transcripts of 167 AR9 genes were detected at the earliest time point sampled. Transcripts of 59 of these genes had maximal levels at 5 or 20 min postinfection and declined afterward. Transcripts of 21 other genes reached a maximal expression level at 40min of infection, but the difference between the expression levels at 20 and 40 min was less than 40%. We refer to all of these transcripts and the corresponding 80 AR9 genes as early (Fig. 4B).

The abundance of transcripts of the remaining 86 genes whose transcripts were detectable at 5 min postinfection significantly increased between the 20- and 40-min time points. We call these transcripts and their genes continuously expressed (Fig. 4C). Genes in this group have both early and late promoters in front of them. In some cases, the kinetics of transcripts of this class was very similar to that of certain early genes (which, however, do not have an upstream late promoter).

One hundred twenty-five late AR9 genes (124 protein-encoding genes and 1 tRNA gene) whose transcripts are absent at 5 min postinfection were detected (Fig. 4D). Transcripts of some late genes were also barely detectable at 20 min postinfection.

**FIG 5:**
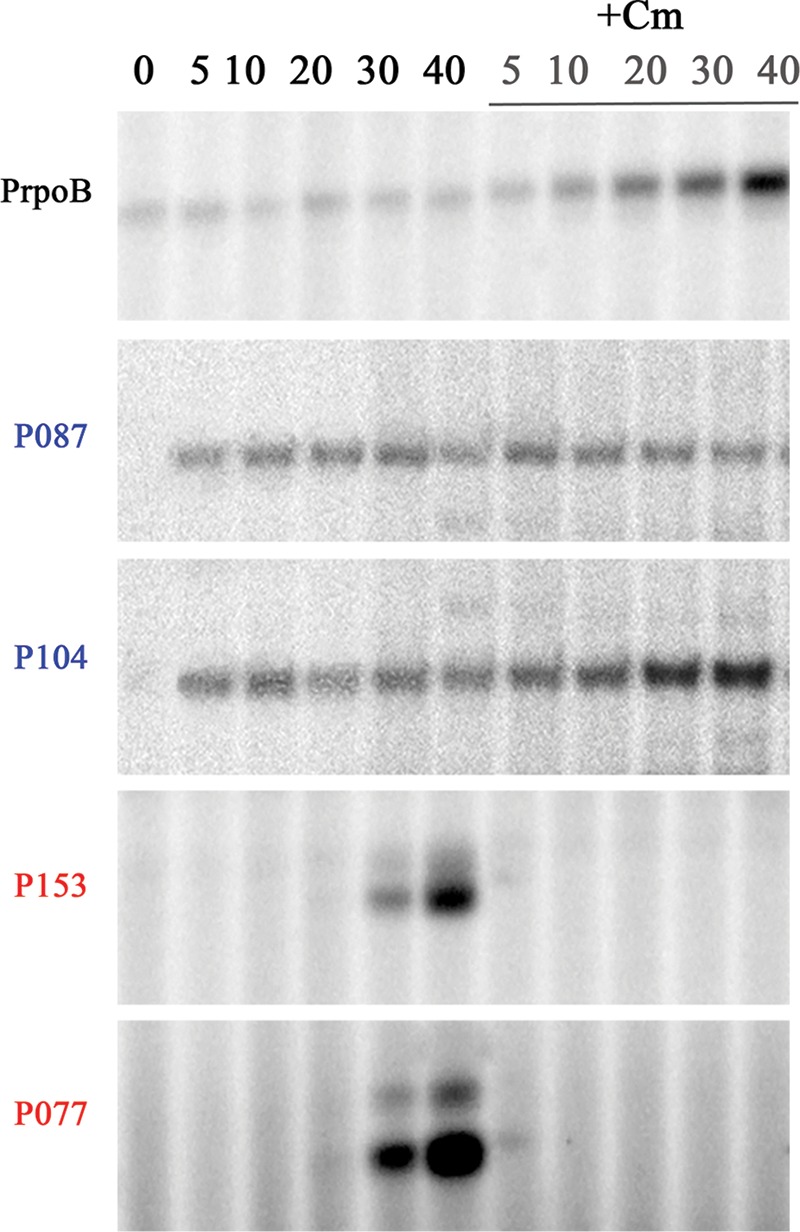
Influence of chloramphenicol on AR9 infection. Extension of primers for AR9 early genes *g087* and *g104* and late genes *g153* and *g077* and the *B. subtilis rpoB* gene on total RNA purified from control cells and from cells treated with 30 μg/ml chloramphenicol (Cm) added 5 min prior to infection. Times of infection (minutes) are indicated at the top.

Accumulation of selected AR9 transcripts during the infection of cells pretreated with chloramphenicol, an inhibitor of translation, was monitored. Accumulation of early transcripts from early promoters P087 and P104 was not prevented by chloramphenicol (Fig. 5). The abundance of host *rpoB* transcript and transcript from an early promoter, P104, was actually increased in chloramphenicol-treated infected cells for reasons unknown. Accumulation of late transcripts (from P153 and P077) was abolished. It therefore follows that early AR9 transcription does not require additional phage-encoded factors other than those that are injected from the virion and must be carried out by vRNAP, while late AR9 transcription depends on the synthesis of phage proteins occurring in the beginning of the infection cycle.

**FIG 6:**
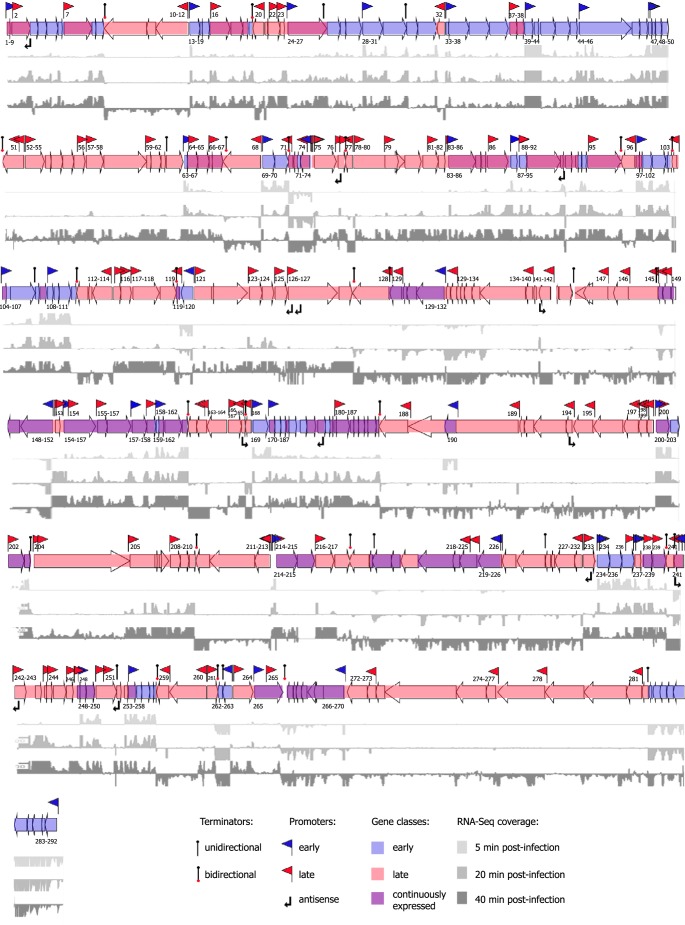
Operon organization of bacteriophage AR9 genes. Shown is a schematic representation of the AR9 genome combined with cDNAs of 5 (light gray)-, 20 (darker gray)-, and 40 (dark gray)-min cDNA libraries mapped on both strands of phage DNA. Coverage for the 5-min time point is shown within a −100-to-100 window size, and that for the 20- and 40-min time points is shown within a −200-to-200 window size. Promoters and terminators are indicated. Early operons are labeled (with numbers marking corresponding genes) below the arrows representing the genes, and late operons are labeled above the arrows representing the genes. The data used to construct this map are available in Tables S2 to S4.

To construct the operon map of AR9, we combined the TSS predictions with calculated levels of expression of neighboring genes. This allowed us to group 292 AR9 genes into 129 operons (Fig. 6). Many predicted long operons contained additional TSSs located in internal intergenic regions, suggesting the existence of numerous “suboperons,” as well as monocistronic transcripts. Many such suboperons/monocistrons belonging to the late expression class were identified within longer primary early operons. For example, late monocistronic transcription units of *g002* and *g007* are located within the early primary operon *g001-g009*. Late suboperon *g037-g038* is located in early operon *g033*-*g038*. As a result of this arrangement, genes preceded by such internal late promoters are continuously expressed. Strong termination of transcription at the ends of all of the predicted operons was evident from RNA-Seq data at the sites of previously predicted terminators (21).

Predicted functions of the products of AR9 genes belonging to different expression classes fit well with patterns observed for other dsDNA phages. Most early AR9 genes encode hypothetical proteins with only a few proteins, for example, DNA gyrase subunit A (gp044) and the N-terminal part of the nvRNAP β subunit (gp105), having a predicted function. Continuously expressed genes encode proteins involved in viral DNA replication and nucleotide metabolism, for example, ribonucleotide-diphosphate reductase complex subunits (gp223-gp224), dCMP deaminase (gp221), RNase H (gp241), uracil-DNA-glycosylase inhibitor (gp104), DNA primase (gp225), DNA polymerase catalytic subunit (gp152), DNA polymerase with a DnaQ-like 3′-5′ exonuclease and a partial catalytic domain (gp132), and NAD-dependent DNA ligase (gp095). Proteins with similar functions are usually encoded by middle genes in other phages. The four nvRNAP subunits (gp089, gp154, gp270, and gp226) are also encoded by continuously expressed genes. Most virion proteins, a putative holin (*g082*), chaperone GroEL (*g228*), an amidase (*g272*), and all of the vRNAP subunits (gp078, gp125, gp145, gp189, and gp264) are encoded by late AR9 genes.

### Expression of *B. subtilis* genes during AR9 infection.

**FIG 7:**
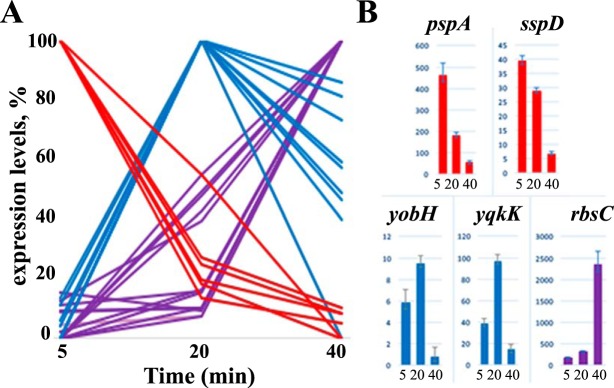
*B. subtilis* 168 genes differently affected by AR9 infection. (A) Host DIGs revealed by RNA-Seq data are presented (downregulated genes are red, and upregulated genes are purple). Genes whose expression is upregulated at the middle of infection (at 20 min) and then downregulated are blue. (B) Validation of RNA-Seq data by RT-qPCR. The expression levels of five selected DIGs were determined by RT-qPCR and normalized to the expression level of the *B. subtilis* housekeeping gene *yoxA* by the ΔΔ*C*_*T*_ method (37).

Since we observed a remarkable decrease in the total number of *B. subtilis* reads during infection, to identify host transcripts with nonlinear abundance changes, differentially expressed genes (DEGs) were identified by normalizing the number of reads that mapped to each of the *B. subtilis* genes to the total number of *B. subtilis* reads. Among 4,175 protein-coding genes of *B. subtilis* 168, only 34 gene transcripts showed significant differences in expression levels according to the Benjamini and Hochberg method (28) (Fig. 7A; Table S5). These host DEGs can be divided into three classes; 16 genes are upregulated at 40 min of infection (purple in Fig. 7A; Table S5), 12 genes are upregulated at 20 min of infection and then their expression levels decrease (blue in Fig. 7A; Table S5), and 6 genes are downregulated at 40 min of infection (red in Fig. 7A; Table S5). Trends from the RNA-Seq data for several randomly chosen DEGs were confirmed by reverse transcription-quantitative PCR (RT-qPCR) (Fig. 7B).

Most of the DEGs affected by AR9 infection encode transporters, transcriptional regulators, and several stress-activated proteins, i.e., general stress protein 39 (*ydaD*), phage shock homolog protein A (*pspA*), and small acid-soluble spore protein D (*sspD*) (Table S5). Most upregulated host genes are located in operons that are under the control of repressors according to the DBTBS database (29). However, there are no common repressor binding sites in front of DEGs, and other genes present in DEG-containing operons are unaffected by infection. The only exceptions are *pstS* and *pstBB*, which are part of the same operon and are both upregulated in AR9-infected cells. Transcription by the host RNAP holoenzyme carrying alternative sigma factors may be affected by AR9 infection, since the abundance of *pspA* and *sspD*, transcribed by the σ^W^ and σ^G^ holoenzymes, respectively, is strongly decreased, while σ^B^-dependent *ydaD* transcript abundance is increased.

In four cases, a difference in expression was detected in intragenic regions that are annotated as Misc_RNA. Misc_RNA_29 is located before phosphoadenosine phosphosulfate reductase (*cysH*), Misc_RNA_45 is located before the gene for the acetolactate synthase large subunit (*ilvB*), Misc_RNA_57 is located before the gene for threonine-tRNA ligase 2 (*thrZ*), and Misc_RNA_60 is located before the gene for a hypothetical protein/efflux transporter (*yxkD*).

## DISCUSSION

In this work, we determined the global pattern of gene expression during bacteriophage AR9 infection of its host, *B. subtilis*. Early AR9 transcripts appear minutes postinfection and do not require protein synthesis in infected cells. It therefore follows that early mRNA is synthesized by the AR9 vRNAP, which is injected into infected cells together with viral DNA. Combined dRNA-Seq and RNA-Seq analyses revealed 41 early promoters that must be recognized by vRNAP. Individual early transcripts have diverse kinetics of transcript abundance during infection. Most early transcripts reach their maximal levels at 20 min postinfection, and then their abundance declines. The transcripts of *g048*, *g109*, *g111*, *g120*, *g169*, *g235*, *g289*, and *g292* are most abundant at 5 min postinfection and may constitute a separate “pre-early” group. The reasons for the diverse behavior of early transcripts may be differences in RNA stability or different affinities of vRNAP for corresponding promoters.

Eighty-seven genes belong to the continuously expressed AR9 gene class. Their transcripts appear early in infection and continue to increase in abundance until the end of infection. Continuous expression is accomplished by a tandem arrangement of early and late promoters preceding genes of this class. Among the continuously expressed gene products are those for DNA replication proteins and four out of five nvRNAP subunits (the remaining subunit, gp105, homologous to the N-terminal fragment of bacterial RNAP β subunits, is a product of an early gene).

One hundred eighteen late AR9 promoters and 125 late genes were identified. Genes encoding all of the lysis proteins and 41 out of 43 previously identified AR9 virion proteins (21) belong to this class; the remaining two—*g084* and *g215*—are continuously expressed genes.

Early genes of many dsDNA phages are the most diverse and encode many hypothetical proteins without an assignable function. Indeed, the percentage of proteins without predicted functions among the products of AR9 early genes is 76%, compared to 45% for continuously expressed genes and 54% for late genes. These proteins may constitute a rich source of potential cytotoxic activities targeting important cellular processes.

To date, no vRNAP has been purified from any phiKZ-related phage virions or infected cells, and the subunit composition of this enzyme is thus unknown. For every phage of the group, four virion proteins homologous to parts of the β and β' subunits of bacterial RNAP can be identified bioinformatically. A recent investigation of essential genes of a member of the phiKZ family, *Salmonella* phage SPN3US (30), identified among the phage virion proteins a small protein (gp244) that corresponds to the C-terminal-most bacterial RNAP β' subunit conserved regions (31). Homologs of SPN3US gp244 can be identified in all phiKZ-related phages (30). Thus, AR9 virions contain five polypeptides that together correspond to the full-length β and β' subunits of bacterial RNAP (gp189/gp078 and gp145/gp264/gp125, respectively). The β- and β'-like subunits of cellular RNAPs require universally conserved additional polypeptides for assembly into a catalytically competent RNAP core (α subunits in bacteria). In bacteria, the core is converted into a holoenzyme by the binding of dissociable σ subunits that allow specific recognition of promoters. Neither AR9 nor other phiKZ-related phages encode homologs of cellular RNAP assembly or promoter specificity subunits. They may therefore rely on nonhomologous viral proteins injected into infected cells together with β- and β'-like subunits of vRNAP or on host proteins to perform these functions. It is also possible that vRNAP polypeptides corresponding to the fragmented cellular β and β' subunits assemble into a functional complex that is capable of specifically recognizing early phage promoters, although this would be an unprecedented situation. Early AR9 promoters are characterized by an extended AT-rich highly conserved consensus element, AAATATATATTAT-(6N)-G^+1^. The vRNAP promoters of all phiKZ-related phages are represented by extended AT-rich highly conserved sequences, and those of two phages studied experimentally, phiKZ and phiR1-37, contain a conserved TSS 6 nt downstream of the consensus elements (19, 32). vRNAP appears to be highly sensitive to deviations from the consensus sequence, at least at some positions; the bioinformatically predicted early promoter AACTATATATTATGATATTG (where conserved promoter positions are underlined) in front of *g057* differs from the consensus sequence in just one position, but *g057* is a late gene and there is no transcription start point associated with the predicted early promoter.

Late promoters are characterized by an AACA-(6N)-TA/G consensus motif and must be recognized by nvRNAP. The phiKZ nvRNAP has been purified and showed to recognize late promoters characterized by a TATG sequence, where the G is the TSS (20). Thus, the promoter recognition properties of nvRNAPs of phiKZ-related phages may differ significantly. Purified phiKZ nvRNAP is capable of specific promoter recognition and contains, in addition to homologs of split β- and β'-like subunits, an additional subunit, gp068, with no similarities to proteins of known function. A distant homolog of phiKZ gp068 is encoded in the AR9 genome, a product of a continuously expressed gene, *g226*. This protein may be responsible for promoter recognition by nvRNAP. The *Yersinia enterocolitica* phiR1-37 phage is, in many respects, the closest relative of AR9; they are the only phiKZ-related phages with uridine-containing genomes, and the β- and β'-like subunits of their RNAPs are most closely related to each other. It also encodes a protein homologous to phiKZ gp068. Unfortunately, a study of the phiR1-37 transcriptome failed to provide a late promoter consensus motif (32).

While independence of viral gene expression from host transcription appears to be a common feature of the phiKZ-related phage group, the transcriptomic architecture of phiKZ-related phages appears to be highly variable. Genes of phiKZ were divided into three transcriptional classes, early, middle, and late. Middle and late genes are transcribed by nvRNAP, and they are characterized by two different promoter consensus sequences, of which only one (late) is recognized by nvRNAP *in vitro* (19, 20). Transcripts of phiR1-37 were classified, by the criterion of their presence at the highest abundance of transcripts during infection, into four classes, early, middle, late, and continuously expressed. The last group was the most abundant, and the levels of these transcripts did not change throughout infection (32), which may mean that the infections were not synchronous. As already mentioned, no promoter consensus elements other than the extensive AT-rich vRNAP motif was identified for phiR1-37.

The AR9 strategy is an elegant one, with the phage relying on two RNAPs with different promoter specificities and times of activity to organize the expression of its genes into three standard temporal classes by relying on continuously expressed genes that are transcribed from both early and late promoters. Variation of the relative strengths of promoters from either class and likely differential stability of individual mRNAs are responsible for diverse kinetics of accumulation of individual continuously expressed transcripts. Functionally, transcripts from this group correspond to middle transcripts of more conventional phages.

The analysis of *B. subtilis* mRNA levels demonstrated that they decrease up to 2-fold at the end of the AR9 infection cycle. We were able to detect 34 genes with significantly changed (upregulated and downregulated) expression levels. The *Y. enterocolitica* response to phiR1-37 infection involved the upregulation of >54% of the DEGs, including ABC transporters; the Cpx system; and phage, cold, and osmotic shock genes (32). A different situation is observed during phiKZ infection, where only a single operon of the *P. aeruginosa* host is upregulated. It encodes proteins of a filamentous prophage, Pf1 (19). The data yet again point to a variety of host responses to phiKZ-related phage infection. More studies are needed to demonstrate the functional significance of host transcription changes during infection with these phages.

phiKZ infection is fully insensitive to rifampin and thus independent of host transcription. Phage AR9 is also capable of infection without the involvement of host RNAP. The decreased phage yield in the presence of rifampin is likely due to *B. subtilis* 168 death caused by rifampin, for, as reported earlier, rifampin affects the cell integrity of this strain irrespective of its effect on host transcription (33). Yet, we cannot fully exclude the possibility that host RNAP transcribes a small number of phage genes and these transcripts contribute to infection efficiency. Alternatively, host RNAP transcription of bacterial genes may stimulate the infection. In this regard, it is interesting that infections with phiKZ-related phages RSL2 and RSF1 were recently shown to be sensitive to rifampin (34), which might point to an even larger diversity of gene expression strategies used by phages of this unusual and underinvestigated group.

## MATERIALS AND METHODS

### Bacterial strains, phage, growth conditions, and one-step growth curve.

Information about phage, bacterial strain, and phage lysate preparation can be found in reference 21. The one-step growth curve experiment was carried out by growing a *B. subtilis* culture in LB medium to an optical density at 595 nm (OD_595_) of 0.6 and infecting it with AR9 at a multiplicity of infection (MOI) of 0.5. Aliquots of infected cultures were withdrawn, and phage titers were determined by the double-layer agar plate method. Burst size was calculated as the ratio of the final amount of liberated phage particles to the initial number of bacterial cells in the infected culture. Where appropriate, rifampin (Sigma) or chloramphenicol (AppliChem) was added to a final concentration of 50 or 30 μg/ml, respectively.

### RNA purification.

*B. subtilis* cells were grown to an OD_595_ of 0.6 and infected with AR9 at an MOI of 10. To synchronize the infection, 2 min after infection, the culture was centrifuged at 5,000 × *g* for 5 min at +4°C and the pellet was resuspended in the same amount of LB medium prewarmed to 37°C. At various time points, 15-ml aliquots of infected cultures were withdrawn, chilled on ice, and collected by centrifugation. Total RNA was purified by the hot-phenol method (35). The RNA pellet was dissolved in 30 to 50 μl of diethyl pyrocarbonate (DEPC)-treated water. RNA samples were treated with RNase-free DNase I (Fermentas) in the presence of RiboLock (Fermentas) for 1 h at 37°C. Samples were then extracted with phenol-isoamyl alcohol-chloroform, ethanol precipitated, and resuspended in DEPC-treated water. The resulting RNA concentration was 3 to 5 μg/μl. RNA concentrations were determined with a NanoDrop spectrophotometer; the quality of RNA was ascertained by agarose gel electrophoresis. The overall levels of rRNA did not change throughout the infection, as determined by visual inspection of agarose gel lanes.

### AR9 DNA purification.

*B. subtilis* cells were collected as described above and lysed in TE buffer (20 mM Tris-HCl [pH 8.0], 1 mM EDTA) with 50 μg/ml lysozyme. DNA was purified with a Thermo Scientific column DNA extraction kit and eluted in 50 μl of elution buffer.

### RT-qPCR and qPCR.

First-strand cDNA was performed with Maxima reverse transcriptase (Fermentas) and random hexamer primers (Fermentas) with 10 μg of total RNA in 25-μl reaction mixtures. Each real-time quantification reaction mixture contained 2 μl of total cDNA, 0.5 μM gene-specific forward and reverse primers, 0.01 μl of fluorescent dye syto13 (Thermo Scientific), 2 U of hot-start DNA polymerase (Evrogen), and 0.2 mM deoxynucleoside triphosphates (dNTPs) in a final volume of 20 μl. As a control, host *yoxA* gene transcript levels were determined with primers *yoxA_F* and *yoxA_R*. For the primers that were used for RT-qPCR, see Table S1. The RT-qPCR conditions were as follows: 3 min of enzyme activation at 95°C, followed by denaturation at 95°C for 20 s and annealing of primers at 60°C for 20 s and extension at 72°C for 20 s. The samples were cooled to 65°C and then heated to 95°C in 0.05°C steps, and the melting curves were determined. The cycle threshold (*C*_*T*_) values of the *yoxA* control transcript were used to normalize the *C*_*T*_ values of selected *B. subtilis* and AR9 transcripts (36). To determine the amounts of AR9 and *B. subtilis* chromosomal DNA during infection, DNA samples were diluted to 20 ng/μl and 2-μl aliquots of total DNA were used for qPCRs. Each reaction was performed three times. The RT-qPCR data were analyzed by the ΔΔ*C*_*T*_ method (37).

10.1128/mBio.02041-16.4 TABLE S1  Sequences of the primers used in this study. Download TABLE S1, DOCX file, 0.1 MB. Copyright © 2017 Lavysh et al. 2017 Lavysh et al. This content is distributed under the terms of the Creative Commons Attribution 4.0 International license.

10.1128/mBio.02041-16.5 TABLE S2  Early promoters of phage AR9. TSSs were predicted by comparison of 20-min TEX-treated and non-TEX-treated libraries. TSSs are in capital letters, and the early promoter motif is red or underlined in cases where two promoters are located in the same area. The coordinate of the TSS is listed in the TSS column, and the length of the 5′ UTR is listed in the UTR column. Promoter classes are defined according to reference 38 as primary, secondary (a promoter upstream of which an alternative primary promoter is found), internal (a promoter within a gene), and antisense. The difference in the expression level calculated for a nucleotide at a position before the TSS and at the TSS is listed in the S. height column. The ratio of the expression level calculated for a nucleotide at the TSS to that at the position immediately upstream is called a step factor, which is listed in the S. factor column. The enrichment factor is calculated as a ratio of the step factors in TEX-treated and untreated libraries. The enrichment factor reflects how 5′ ends respond to TEX treatment (an increase corresponds to a number >1, and a decrease corresponds to a number <1). Download TABLE S2, DOCX file, 0.02 MB. Copyright © 2017 Lavysh et al. 2017 Lavysh et al. This content is distributed under the terms of the Creative Commons Attribution 4.0 International license.

10.1128/mBio.02041-16.6 TABLE S3  Late promoters of phage AR9. TSSs are in capital letters, the late promoter motif is blue. NA is in the UTR column if the transcript is leaderless, antisense, or internal. TSSs were predicted by a comparison of 20-min TEX-treated and untreated libraries; for a few promoters that were predicted manually from an untreated 40-min library, NA is entered in the S.factor and enrichment columns. Genes with promoters that have either different distances between the conserved AACA tetranucleotide and the TA/G element at the TSS or carry one or two nonconsensus bases are red. Genes whose promoters lack consensus motifs are dark red. Download TABLE S3, DOCX file, 0.05 MB. Copyright © 2017 Lavysh et al. 2017 Lavysh et al. This content is distributed under the terms of the Creative Commons Attribution 4.0 International license.

10.1128/mBio.02041-16.7 TABLE S4  Transcription classes of AR9 genes. For each gene, the total number of mapped nucleotides was calculated and normalized to the total number of mapped reads for each library (FPKM levels). The normalized expression levels across three time points (5, 20, and 40 min postinfection) are color coded blue for high and yellow for low expression levels (FPMK). Genes are classified as early (blue), continuously expressed (green), and late (red). Download TABLE S4, DOCX file, 0.1 MB. Copyright © 2017 Lavysh et al. 2017 Lavysh et al. This content is distributed under the terms of the Creative Commons Attribution 4.0 International license.

10.1128/mBio.02041-16.8 TABLE S5  *B. subtilis* 168 genes with significantly changed expression levels during AR9 infection. Genes are colored according to their transcription kinetics shown in Fig. 7. If a gene is involved in a KEGG pathway, the name of the pathway is listed. If there is information about the regulation of operon expression, it is listed in the regulated-by column. Download TABLE S5, DOCX file, 0.02 MB. Copyright © 2017 Lavysh et al. 2017 Lavysh et al. This content is distributed under the terms of the Creative Commons Attribution 4.0 International license.

### Primer extension and sequencing reactions.

Five micrograms of total RNA and 1 pmol of [γ-^32^P]ATP end-labeled gene-specific primer were combined in 3 μl of Maxima reverse transcriptase buffer (Fermentas). The samples were heated to 65°C for 5 min and immediately cooled to 4°C, and then RiboLock (Fermentas), dNTPs, and Maxima reverse transcriptase were added according to the manufacturer’s instructions to a final volume of 20 μl. The samples were incubated at 50°C for 1 h and heated in an equal volume of LD buffer (80% formamide, 1 mM EDTA, 0.025% xylene cyanol, 0.025% bromphenol blue) for 10 min. The reaction products were separated on a 6% denaturing urea–polyacrylamide gel and visualized with a Typhoon PhosphorImager (GE Healthcare). Sequencing reactions were carried out with the USB Thermo Sequenase Cycle Sequencing kit and PCR products with the same primers as the ones used for the primer extension reactions.

### dRNA-Seq library preparations.

Library preparations were done as described by Sharma et al. (38). Equal amounts of total RNA were used to generate cDNA libraries by random priming/RT. For depletion of processed transcripts, one part of the RNA was treated with TEX exonuclease (Epicentre). cDNA libraries were constructed by *vertis* Biotechnology AG, Germany. Sequencing of cDNA libraries was performed on a HiSeq2000 sequencer (Illumina). In total, 4 million to 7 million cDNA reads were sequenced for each cDNA library. Four libraries were created (5 min, 20 min, 20 min +TEX, and 40 min).

### TSS prediction by dRNA-Seq analysis and differential gene expression analysis.

After adaptor clipping and quality trimming with cutadapt (https://doi.org/10.14806/ej.17.1.200), the reads were mapped to the AR9 genome sequence (KU878088) and the *B. subtilis* 168 genome sequence (NC_000964.3) with READemption version 0.3.0 (39) and segemehl version 0.1.7 (40). For data visualization, graphs representing the number of mapped reads per nucleotide were calculated and normalized to the total number of reads. The resulting wiggle files were visualized with the Integrated Genome Browser, version 6.5.3 (41). TSSs were predicted with TSSPredator version 1.05 (42). The numbers of reads mapped to a gene in the sense and antisense directions were calculated. DEGs (exhibiting at least 2-fold changes in expression) were screened via the cufflinks and cuffdiff algorithms installed in the Galaxy platform (43). The associated *P* values were adjusted for multiple testing by the Benjamini and Hochberg method to keep the false-discovery rate at <5% (*P* < 0.05) (28).

### Accession number(s).

The sequencing data have been deposited in the NCBI Gene Expression Omnibus (44) and are accessible through GEO Series GenBank accession no. GSE87573 (https://www.ncbi.nlm.nih.gov/geo/query/acc.cgi?acc=GSE87573).
