# Supplementary figures and images for: Transcription Profiling of Bacillus subtilis Cells Infected with AR9, a Giant Phage Encoding Two Multisubunit RNA Polymerases
Source: mBio. 2017 Feb 14;8(1):e02041-16. doi: 10.1128/mBio.02041-16 (PMC5312081; doi:10.1128/mBio.02041-16)

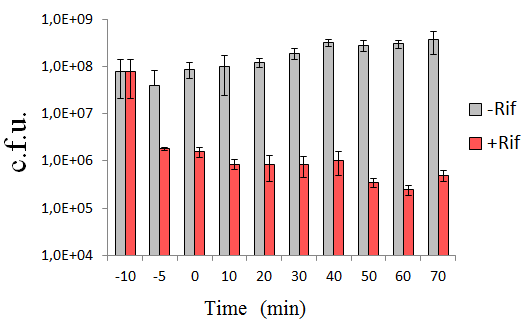

Supplement: FIG S1 [file mbo001173180sf1.tif]

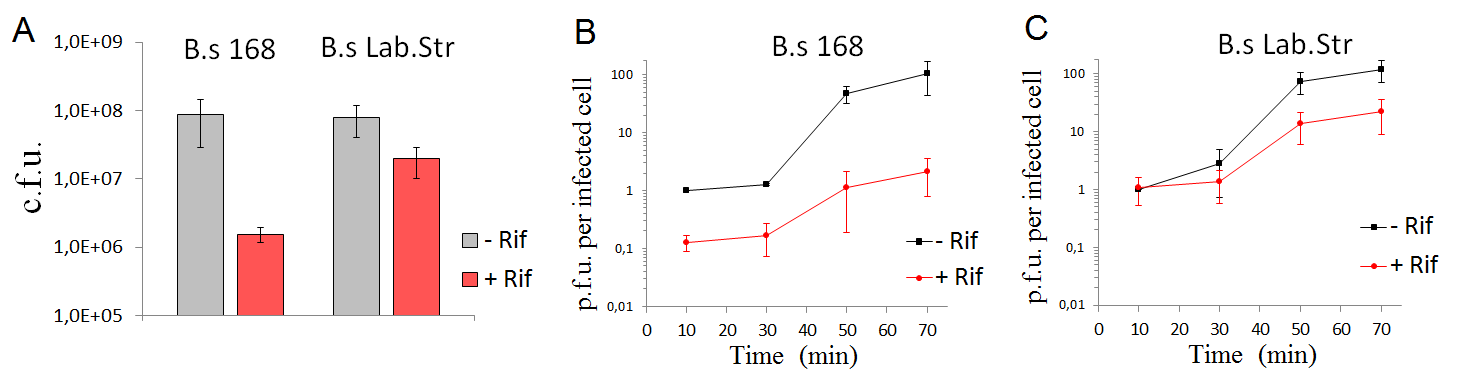

Supplement: FIG S2 [file mbo001173180sf2.tif]

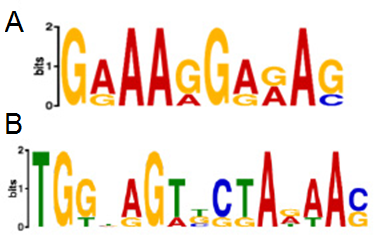

Supplement: FIG S3 [file mbo001173180sf3.tif]
