# Supplementary material for: Transcription Profiling of Bacillus subtilis Cells Infected with AR9, a Giant Phage Encoding Two Multisubunit RNA Polymerases
Source: mBio. 2017 Feb 14;8(1):e02041-16. doi: 10.1128/mBio.02041-16 (PMC5312081; doi:10.1128/mBio.02041-16)
Supplement: TABLE S1 [file mbo001173180st1.docx]

**Table S1. Primer sequences used in this study.**

| **name** | **sequence (5’-3’ orientation)** | **name** | | **sequence (5’-3’ orientation)** |
| --- | --- | --- | --- | --- |
| for RT-qPCR, RT-PCR experiments: | | | | |
| glpT_f | AGCCAAGAAGACGATGGCAA | pspA_f | | AAGGCACTTGATCAGGCCAA |
| glpT_r | CGCCATTATGGGCTTTGTCG | pspA_r | | TTTTCGCTTTTAACGCCGCA |
| lmrA_f | TCCTCATACACAGAGGCCCA | yoxA _f | | CCTTTGTTCATGACCGTCGC |
| lmrA_r | GGAGCTCTCCTGCCAGTTTT | yoxA _r | | TCCCCATGTGCAGAAGCAAT |
| rbsC_f | CTGTTGGCGCTATTCTTGCC | g044_F | | TTACCTTCTATTCTTCCAATGATATTAG |
| rbsC_r | ACACAATCATCCCCGCAACT | g044_R | | GCCTTTTATTAATAATTACTCCACCAG |
| ydjM _f | TCATCAGATGCCAGTGCGAA | g078_F | | ATCTGATATTGAATCATATAGTCTTGTTG |
| ydjM _r | TCGCACAATCCCAATGTCCA | g078_R | | TCCAGCACCCATTAGATATACATAC |
| yqkK_f | TGAAAGATGGTGTGCTGCCT | g283_f | | TGGACAGTGGGTGGTAATCC |
| yqkK _r | GGCATCGATTGAGAAACGGC | g283_r | | ACCAGTAATTGCATCTGGTTTAACT |
| yobH_f | CTCCCTCCTATCCCCCACAT |  | |  |
| yobH _r | AAGCTTGCACTTGACCTGGA |  | |  |
|  |  |  | |  |
| for primer extension and sequencing reactions: | | | | |
| g120_pe | ATTCATAATTGATTCCAAAGTATTC | g077_pe | ACTTACACGAATTGCTTCATT | |
| g121_pe | CCAGATCTTAATAAAACTTAGGTTTC | g077_pcr | TCTTTCTATGTTCCGAATACC | |
| g152_pe | TGGATATGAATTTACATAATTTTTAG | g233_pe | GGAGTTCACTCTTAAATATATCATC | |
| g153_pe | GTGAGTAAGTTGAAGAATGATGAC | g233_pcr | GATTATTCATCTTTGGTACTAACAT | |
| g087_pe | ACACTCTCTGGTTGAATCTCTACG | rpoB_pe | GAAAACCACATCCAAAAACAAATACC | |
| g087_pcr | TCCTATCCGGTATTGATCTCTCAG | | | |
| g104_pe | CCTGTTTCTTTTTCAATGATGTC | | | |
| g104_pcr | TCTATAAATTTGTTCTAGAACCCAC |  |  | |
