# Supplementary material for: Transcription Profiling of Bacillus subtilis Cells Infected with AR9, a Giant Phage Encoding Two Multisubunit RNA Polymerases
Source: mBio. 2017 Feb 14;8(1):e02041-16. doi: 10.1128/mBio.02041-16 (PMC5312081; doi:10.1128/mBio.02041-16)
Supplement: TABLE S2 [file mbo001173180st2.docx]

**Table S2. Early promoters of phage AR9.**

| Gene | Sequence -50 nt upstream + TSS (51nt) | TSS | Strand | UTR | Class | S. height | S. factor | Enrichment |
| --- | --- | --- | --- | --- | --- | --- | --- | --- |
| *g001* | tacggttaataatattattaattcatttttcaattatatattataaagatG | 66 | + | 33 | Primary | 1353 | >100 | 2,51 |
| *g013* | aagtaattgttcaaagtttatttaaaaaatgaaatttatattataataaaG | 11208 | + | 34 | Primary | 334 | >100 | 0,96 |
| *g024* | aaaaatatgattgaaatttgacctattttataaatttatattattatataG | 17266 | + | 109 | Primary | 19 | >100 | 0,67 |
| *g028* | aatataataagaagaaattaaaataactttaaagtttatattataaaaatG | 21888 | + | 50 | Primary | 305 | >100 | 0,92 |
| *g033* | tagctatattattatgtttaaaaaaatttaaaaatatatattatacttttG | 26979 | + | 41 | Primary | 165 | >100 | 1 |
| *g039* | tctcccttatataaggtttaaaaagaaaaataaatagatattataataatG | 31858 | + | 31 | Primary | 1158 | >100 | 0,4 |
| *g044* | tagattagaacttatgaataatttaaaaaataaagttatattattatcAtA | 35064 | + | 200 | Primary | 188 | 8,23 | 0,35 |
| *g047* | ttgctaattaaacgtattaaaatgattaactaaacatatattatcttcctG | 39475 | + | 30 | Primary | 204 | 41,8 | 0,81 |
| *g048* | aaaataaaaaaccgtattaaactacctaagcaaatatatattataatcttG | 39727 | + | 31 | Primary | 1871 | >100 | 1,37 |
| *g063* | gttatttaataatacgactaatatgtttttaaaatatatattatatttttG | 51909 | + | 55 | Primary | 474,5 | >100 | 0,94 |
| *g069* | ttaggagaaaaccgtatcaaaatttttttataaatatatattataattggG | 56778 | + | 40 | Primary | 1986 | >100 | 1,52 |
| *g074* | tgtgaagaaaaaattatttaaatcagtcattaaatatatattattaaaaaG | 59760 | - | 28 | Secondary | 64 | 2,05 | 0,47 |
| *g083* | catttaattgtcattaccttaaatgaaatataagaatatattattAatttG | 68276 | + | 26 | Primary | 295 | 3,95 | 1,55 |
| *g087* | ttctcattatcttttttccaatttattttttagatatatattataaaaaaG | 72079 | + | 99 | Primary | 914 | >100 | 1,38 |
| *g097* | actttttattaaattgttctaatagaaatataaatatatattattatactG | 79931 | + | 27 | Primary | 2174 | >100 | 1,07 |
| *g104* | catgcaaattataatatttaaaaagttatgaaaatatatattatttataaG | 82726 | + | 121 | Primary | 4632 | >100 | 1,9 |
| *g108* | atagttatttaccgtattaaaaatatttttcaagcatatattatattcctG | 85593 | + | 38 | Primary | 1141 | >100 | 0,77 |
| *g120* | tagaatgtttgtaccgtattaaaatataaaactaagtatatattatatatG | 94722 | - | 27 | Primary | 1612,5 | >100 | 0,71 |
| *g132* | tatgtggatattaaattcacataaccactttaaatatatattatataaatG | 110170 | - | 99 | Primary | 163 | 28,17 | 0,7 |
| *g152* | tatatttaattgtttaaataaaactctttttaaaaatatattattatattG | 127150 | - | 78 | Primary | 883 | 41,14 | 1,97 |
| *g154* | ttaagatattaaaaaacataaaaagtgatgtaaatatatattattaacttG | 127757 | + | 48 | Primary | 547,5 | 10,78 | 1,58 |
| *g157* | atagttgtaaaaaacaattaaaacagagacaaaatatatataataaaaaaG | 131828 | + | 124 | Primary | 58 | 20,33 | 0,33 |
| *g159* | caactatttgatgtatatcaaaataaaagataaatttatattataataGtG | 133407 | + | 26 | Primary | 5078 | 43,67 | 1,18 |
| *g169* | aaatttcagagacgtattaaaattttagatgaaatatatattattaaagtG | 139425 | + | 37 | Primary | 701,75 | >100 | 0,69 |
| *g170* | taatgcctttcttttttttattgaaaaaattaaatatatattataaaaaaG | 140433 | + | 27 | Primary | 967 | >100 | 1,33 |
| *g190* | aataggacaatccgtatcgaagactctcgttaaatatatattataatAgtA | 152042 | - | 27 | Primary | 263 | >100 | 3,82 |
| *g200* | tatagtttggagaaatttcaaactgaaaaataaatatatattattttattG | 164233 | + | 78 | Primary | 2229 | 4,45 | 0,17 |
| *g214* | attgttctaagtaaaagaaaactcatttttcaaatatatattataagaatG | 181724 | + | 340 | Primary | 264 | 3,06 | 3,87 |
| *g226* | aatctcaaaaaagagttgcaaagaaagagaaaaatagttataatggagaaG | 195839 | - | 31 | Primary | 438 | 40,82 | 1,95 |
| *g226* | ttagaaagaagaaaaagtaatataacactttaaatatatattataaaaaaG | 196022 | - | 214 | Secondary | 29 | 6,8 | 1,22 |
| *g234* | atttttaataaccgtattaaaatattaaaagaaatatatattatataattG | 201716 | + | 33 | Primary | 331 | >100 | 0,69 |
| *g237* | tattttttaagtaaaattaaaatattataacaaatatatattatataattG | 204069 | + | 44 | Secondary | 3 | 1 | 0,51 |
| *g241* | ttaattattattaataacatatacatgtttcaaatatatattattataatG | 207106 | - | 29 | Primary | 205 | 14,67 | 0,82 |
| *g248* | cctataatcctaaaagattataggagtttttaaatatatattatttctttG | 210848 | + | 191 | Primary | 30 | 31 | 0,67 |
| *g253* | ttatcttcataccgtattatagtaatatttcaaatatatattatattaatG | 213869 | + | 119 | Primary | 85 | 43,5 | 0,64 |
| *g263* | tattctattgttcatagtaaatgtaaattttaaatatatattatattaatG | 220691 | - | 33 | Primary | 51,33 | 3,57 | 1,5 |
| *g265* | ttttccatttaataattaagtaccattaaacaaatatatattataattGtA | 222051 | + | 28 | Primary | 24 | 1,69 | 0,62 |
| *g270* | gaaagtctttgcttattctattataaaaattaaatatatattatttttatG | 227681 | - | 161 | Primary | 173 | 25,71 | 2,52 |
| *g292* | aaatatatattataattgtG | 251023 | - | 79 | Primary | 193 | 25,13 | 0,7 |
