# Supplementary material for: Transcription Profiling of Bacillus subtilis Cells Infected with AR9, a Giant Phage Encoding Two Multisubunit RNA Polymerases
Source: mBio. 2017 Feb 14;8(1):e02041-16. doi: 10.1128/mBio.02041-16 (PMC5312081; doi:10.1128/mBio.02041-16)
Supplement: TABLE S3 [file mbo001173180st3.docx]

**Table S3. Late promoters of phage AR9.**

| Gene | Sequence -50 nt upstream + TSS (51nt) | TSS | Strand | UTR | Class | S.  height | S.  factor | Enrichment |
| --- | --- | --- | --- | --- | --- | --- | --- | --- |
| *g007* | ttattatcagaaataaaaaagagaaaataacaacagatgaacatacaagtG | 3551 | + | 32 | Primary | 702 | >100 | 1,5 |
| *g012* | tttattataatataaatttcattttttaaataaactttgaacaattacttA | 11157 | - | 20 | Primary | 19 | >100 | 0,94 |
| *g016* | atgaatgataaattttcagtaaaatttaaaaatattgataacaaggtattG | 12416 | + | 114 | Primary | 28 | 29 | 0,12 |
| *g020* | gatccttaaaaaatgagcagtttttataacattgaattaaacaaaatatcA | 15872 | - | 23 | Primary | 13 | 7,5 | 2,65 |
| *g022* | ttaattatagaaaaattaagaagaaaaaaagaaaaggttaacagtaaattA | 16125 | + | 29 | Primary | 87 | 88 | 2,22 |
| *g023* | attgaattgctttagttatattatcaactatgatgataaaacaatatattA | 16626 | + | 263 | Primary | 22 | >100 | 1,06 |
| *g037* | tatgccatggcattgtcattacaaaatctaagaattaacaacatgaagatG | 30926 | + | 25 | Primary | 37 | >100 | 0,54 |
| *g051* | atttttatgtataaaaaattagatagtgaattaaagaataacaaagaattA | 40957 | - | NA | Internal | 5 | 3,5 | 0,23 |
| *g051* | tgtcagtttataataaaatgtttactatgtatattaggaaacattttattA | 42089 | - | 29 | Primary | 6 | >100 | 0,28 |
| *g052* | tttaatatcttaataaaatgtttcctaatatacatagtaaacattttattA | 42129 | + | 28 | Primary | 12 | 5 | 0,84 |
| *g056* | tgggaatgccttctcatattattaatcgtgaagatctagaacaaataattA | 45237 | + | NA | Internal | 4 | >100 | 0,85 |
| *g059* | gctgaaaaggtatactttgatttcaagaaagactaccaaaacaatttaatA | 49366 | + | 246 | Primary | 30 | 3,14 | 1,29 |
| *g064* | ttttaggaggtattttttaaaaaagttatgaaaaaagataacattttaaaG | 51987 | + | NA | Internal | 11 | 1,02 | 0,82 |
| *g068* | tttgatacggttttctcctaactttttatgagaagttcaaacactcaattG | 56698 | - | 30 | Primary | 400 | >100 | 1,05 |
| *g071* | tgataaaccattgccttggaatggtgttccagttaagaaaacaagtatatA | 58501 | - | NA | Internal | 57 | 3,04 | 0,09 |
| *g075* | atgtattttttataaataataaaagatatcattttacataacaacatgttA | 59871 | + | 169 | Secondary | 1071 | 22,21 | 1,61 |
| *g075* | tgtttatttttttatttgcttaaaaaaattagagaatggaacattatattA | 60010 | + | 30 | Primary | 61 | 8,63 | 0,71 |
| *g076* | cgccacctaaaaaggttatttttagagcagtaaaagataaacaagataacT | 61654 | + | NA | Internal | 4 | >100 | 0,30 |
| *g077* | acttatctagtctataattaattgttgtcttcttcaataaacaatatattA | 62413 | - | 24 | Primary | 215 | 8,41 | 0,80 |
| *g078* | tccaatatgtgatataatatattgtttattgaagaagacaacaattaattA | 62449 | + | 27 | Primary | 18 | 4 | 0,64 |
| *g079* | cttttatttaattctttaaaaaagagctgacaacactaaaacaaaaatatA | 64330 | + | 25 | Primary | 32 | 6,33 | 0,64 |
| *g081* | catacggtttaacagccaaatatgatattccatttattaaacaaataattA | 66651 | + | 44 | Internal | 5 | >100 | 0,81 |
| *g086* | tgtattcactttttaatagaaaaaatttagagaattcctaacaagtaattG | 70697 | + | 35 | Primary | 262 | >100 | 0,19 |
| *g088* | aattaaggaataaattcttaaaaaataaaaagaatgaaaaacaaaagaatG | 72685 | + | 52 | Primary | 259 | >100 | 0,64 |
| *g090* | cttcctctcaaaattatgtcttttttattacaggaaataaacatattaatA | 75478 | - | NA | Antisense | 3757 | 49,17 | 1,05 |
| *g096* | attgatcagtataataatatatatttatatttctattagaacaatttaatA | 79887 | - | 28 | Primary | NA | NA | NA |
| *g103* | tatattttcataactttttaaatattataatttgcatgtaacaaatatatA | 82663 | - | 162 | Primary | 88 | 3,84 | 1,1 |
| *g114* | atattatgatttaacttaattatatgttttttattagaaaacatataattA | 89691 | - | 38 | Primary | 20 | 11 | 0,95 |
| *g115* | acttaattaaatttattaattatatgttttctaataaaaaacatataattA | 89725 | + | 68 | Primary | 7 | >100 | 1,85 |
| *g116* | ttctaattgattttaataaaaaattaatggaagatacaaaacataataatA | 90308 | + | NA | Internal | 189 | 95,5 | 5,62 |
| *g117* | tataaatcaatttattagattttatattagagagatctaaacaaaaaattA | 90955 | + | 37 | Primary | 22 | 2,69 | 1,06 |
| *g121* | atataatatatacttagttttatattttaatacggtacaaacattctattA | 94775 | + | 27 | Primary | 7 | 8 | 0,77 |
| *g123* | tgaaaatctacattcttttttatattgaagagaattactaacaaattcatA | 98069 | + | 44 | Primary | 381 | >100 | 0,58 |
| *g125* | cctatatattaaattatatataggtttacttattattaaaacataatattG | 99678 | + | 31 | Primary | 47 | >100 | 2,21 |
| *g126* | aagaataattaagtactaaaattttcttatttgtaaaagaacatttaattA | 100487 | + | 34 | Primary | 77 | 78 | 2,58 |
| *g128* | aacatgctttattttttttttatttttttcataaagaaaaacatttaattA | 106667 | - | 29 | Secondary | 22 | 23 | 0,55 |
| *g129* | gcgtctaaagaaatctcaagttaaaaataaagaacagataacaagtaaatG | 107551 | - | 109 | Primary | 1086,67 | 48,25 | 0,33 |
| *g134* | aatttttctttggaaatattaaaaataaagaagatttagaacaagctattA | 111066 | - | 172 | Primary | 14 | >100 | 0,17 |
| *g140* | aaataatttatattcttgtaactaaaaaaattatttaaaaacatgaaaatG | 115556 | - | 30 | Primary | 26 | >100 | 1,22 |
| *g142* | tttaatctttttttatttttagagaattaattacaataaaacataattatA | 115851 | + | NA | Antisense | 278 | >100 | 2,2 |
| *g142* | cacagtaataatacatacttgaaaatctttttgatacggaacattatattA | 116648 | - | 28 | Primary | 19 | 20 | 0,4 |
| *g145* | ccttcatattttttttttgttcatttctcttaaaccgttaacaaattattA | 123185 | - | 22 | Primary | 18 | 10 | 0,76 |
| *g146* | ttaatattatttctttttaagggaatacagtgtcaaataaacaaataattA | 120207 | - | 25 | Primary | 18 | 10 | 0,76 |
| *g147* | gtcatagtcagtttaataaaaataatcgaacaatcacaaaacataataatA | 121466 | - | 24 | Primary | 24 | 4,43 | 0,69 |
| *g149* | ttattctgatatttctaaaaagactggggtaaaatatcaaacagtacaatA | 123887 | - | NA | Internal | 7 | 2,75 | 0,23 |
| *g153* | atcaatataataatatatttttaaaaagagttttatttaaacaattaaatA | 127198 | + | 62 | Secondary | 53 | 7,63 | 0,94 |
| *g153* | tatttaaacaattaaatataaaagtatttatttcaaataaacattaaattA | 127231 | + | 29 | Primary | 231 | 3,78 | 0,68 |
| *g155* | tttttacatattaatgtataaataaataaaaatatagataacaaaatatcA | 129798 | + | 25 | Primary | 36 | 37 | 0,24 |
| *g158* | gttatatttttgtagatagatatgttactgaaattacagaacatatatttG | 132624 | + | 208 | Primary | 17 | 9,5 | 0,25 |
| *g164* | attctcttcatactaaaagaaataaagaatacaattttaaacaattaaatA | 136579 | - | 38 | Primary | 218 | 28,25 | 0,81 |
| *g164* | gataattataaaataaataattttataattaattttttaaacaatgctttA | 136708 | - | 167 | Secondary | 50 | 26 | 1,93 |
| *g166* | agttattatttacatgataaactttgattcatttgttgaaacatactattA | 137867 | + | 40 | Primary | 6 | >100 | 0,67 |
| *g167* | agatgaggataaggtctttgttatctctcaaaatccagaaacaaatgattA | 139024 | - | NA | Antisense | 42 | 4,5 | 0,27 |
| *g168* | tacgtctctgaaatttttaatttttttatgtgaataatcaacatttagttA | 139340 | - | 33 | Primary | 455 | >100 | 0,57 |
| *g180* | taaaaattgatttttctataactaaaatagagatttagtaacaatttatcA | 144481 | + | 17 | Primary | 736 | >100 | 0,62 |
| *g188* | gaataattttttattgataaacaaattggatttcaactaaacaaaataatA | 149101 | - | 26 | Primary | 43 | 15,33 | 1,05 |
| *g189* | ggataaaaccttatctttttatttttaatttcttatgtaaacaaatagatA | 155790 | - | 26 | Primary | 85 | 18 | 6,57 |
| *g194* | aaagggtatgcttattttttatatttttactaaattaaaaacaattatttA | 159098 | - | 18 | Primary | 46 | 47 | 2,37 |
| *g195* | ctatagctgtctaatattattttatcgcattattaaagtaacattttattA | 160380 | - | 29 | Primary | 59 | >100 | 5,95 |
| *g197* | gacgatgatgaaatattcactttgataatcgattgatagaacaaaaaattA | 163188 | - | 26 | Primary | 37 | >100 | 3,56 |
| *g199* | aaattttaaaaagaattaataatagttagaacaaacgagaacaacttattA | 164112 | - | 30 | Primary | NA | NA | NA |
| *g200* | aactattattaattctttttaaaatttaaaagaatagataacagttatatA | 164186 | + | 125 | Secondary | 636 | >100 | 1,78 |
| *g204* | aatggaagttaataatcattaattttaatgaaaataattaacaaaaaaatA | 167158 | + | 153 | Secondary | 30 | 4 | 0,08 |
| *g204* | tctattattaatattaaaatattaattttttattatataaacaatattatA | 167250 | + | 61 | Primary | 24 | 3 | 0,39 |
| *g205* | aaatgcaaagtattcttaaacaaattaatgagaacagcaaacaaggaaatA | 172936 | + | 170 | Primary | 366 | >100 | 6,53 |
| *g208* | aatacaattctaaattcctaacttttaaagaatatgcctaacagattaatA | 175529 | + | 30 | Primary | 992 | >100 | 0,21 |
| *g213* | tataatatatatttgaaaaatgagttttcttttacttagaacaattaaatA | 181669 | - | 26 | Primary | NA | NA | NA |
| *g214* | tggttatatttttttatttttttcttttcctcctttgttaacaaataattA | 182012 | + | 53 | Primary | 39 | >100 | 0,97 |
| *g216* | cactagtttagatttatattaaaattataaatacaaataaacatataattA | 184406 | + | 45 | Primary | 177 | 23,13 | 0,33 |
| *g225* | tatatgctttaaagaaaaaattagacttgtctgaatcagaacataaaattA | 193580 | - | NA | Internal | 130 | 15,44 | 3,35 |
| *g225* | taaattttttattttgaagatcttttaaagagataatttaacagttttttA | 194327 | - | 27 | Primary | 201 | 26,13 | 1,19 |
| *g232* | aaatattgtttattattttttataatttggagaattgttaacatttcaatA | 200780 | - | 36 | Primary | 641 | 18,32 | 1,1 |
| *g233* | ttgaaatgttaacaattctccaaattataaaaaataataaacaatattttA | 200832 | + | 35 | Primary | 17 | 6,67 | 0,71 |
| *g236* | taaaagatggttttatttatattccatcagttataaaagaacagaataatA | 203501 | + | NA | Internal | 53 | 5,08 | 0,58 |
| *g237* | ataatataatagactttaaactacttatcaatttaaaaaaacaaataaatA | 203990 | + | NA | Secondary | 54 | >100 | 1,82 |
| *g237* | atttttttttatattttttaagtaaaattaaaatattataacaaatatatA | 204058 | + | 28 | Primary | 3550 | 11,09 | 0,47 |
| *g238* | catctgtcactaattatctaaaatttttagtaaattagtaacaaaagattG | 204622 | + | 25 | Primary | 311 | 45,43 | 1,8 |
| *g239* | ttatcaatacttagagaataaaaagtttatttaaacataaacaatcgattA | 205185 | + | 51 | Primary | 24 | 1,44 | 0,31 |
| *g241* | ttatgaaatattatattgtttattttaattattattaataacatatacatA | 207130 | - | 53 | Internal | 13 | >100 | 0,92 |
| *g241* | tcaattaataggtaaacgattaaaagcttttgataaatcaacaaataattA | 206929 | - | NA | Internal | 21 | 4 | 0,34 |
| *g242* | atttgaaacatgtatatgttattaataataattaaaataaacaatataatA | 207172 | + | 32 | Primary | 4 | >100 | 0,61 |
| *tRNA* | tatttctcttttttattttaaaaattaccctgcatttcaaacaatttaatA | 207979 | + | NA | Primary | 73 | 37,5 | 2,02 |
| *g244* | tttaatttcatcattttttataaaaataatcttacatttaacatataaatA | 208874 | + | 146 | Primary | 2101 | >100 | 4,82 |
| *g247* | taatagattaagtgagtcattatcctctaattagtttataacaataaattA | 210346 | + | 23 | Primary | 51 | 52 | 0,99 |
| *g251* | aatgactaagtagataattcatggggaatttaaaataagaacaatcttatA | 212165 | + | 65 | Primary | 5 | 1,63 | 0,25 |
| *g251* | tgaaatatgacttttttcttcctactcattttataaaagaacaatatgatA | 213130 | + | NA | Internal | 748 | >100 | 2,06 |
| *g259* | gaagataaggttatttatgaggaagtttaaagaatagtaaacatttatttG | 216836 | - | 29 | Primary | 6091,5 | >100 | 1,69 |
| *g260* | ttaattttttttatcattttcacatttcctccatttctgaacataaagatA | 219097 | - | 24 | Primary | 197 | 99,5 | 1,26 |
| *g261* | tttctagttagatatctctggacggtagagaatattaagaacaatttaatA | 218639 | + | 491 | Primary | 5 | >100 | 5,30 |
| *g264* | cattaatataatatatatttaaaatttacatttactatgaacaatagaatA | 220741 | + | 32 | Primary | 28 | >100 | 0,49 |
| *g265* | ttttattttagttttccatttaataattaagtaccattaaacaaatatatA | 222040 | + | 39 | Internal | 10 | 11 | 0,51 |
| *g277* | ttcatttacattattatttctagttcttaagatgaaaagaacatatagatA | 236823 | - | 34 | Primary | 146,33 | 2,94 | 2,58 |
| *g278* | ttgaacgaaaatttatcactagtgttgccttacttaaataacaatatattA | 239818 | - | 74 | Primary | NA | NA | NA |
| *g281* | tcattattatcataacatgaatatattatggtaatcctgaacaaataaatA | 245635 | - | 26 | Primary | 12 | >100 | 0,77 |
| *g032* | tttagtatcaaaagtataatatatatttttaaatttttttaaacataataA | 26937 | - | 22 | Primary | 4 | >100 | 2,12 |
| *g057* | agttgaatttttattttataataatagtggaaaacaaaaaactatatattA | 45844 | + | 103 | Primary | 37 | 38 | 2,98 |
| *g066* | cttatactatcggtaagaaagaaagtgagtacacattcttaacatacgatA | 53463 | + | 54 | Primary | 34 | 35 | 1,2 |
| *g074* | gttccattctctaatttttttaagcaaataaaaaaataaacaaaattaatA | 59951 | - | 219 | Primary | 51 | 9,5 | 1,08 |
| *g074* | atgtaaaatgatatcttttattatttataaaaaatacataaccaattaatG | 59809 | - | 77 | Secondary | 60 | >100 | 0,54 |
| *g076* | taattataccaccaaatccttgtttttcctattttttaacaaaacaatgcA | 61440 | + | 46 | Primary | 55 | >100 | 0,9 |
| *g095* | gtataatataggaaataatttcaatataaatgaaatataacaacaaaaaaT | 76941 | + | 50 | Primary | 1232 | 31,8 | 0,93 |
| *g125* | agcgaggcggttatgctatctttgttggattcttaattaacaatattaatA | 99510 | + | 208 | Primary | 4 | 1,5 | 0,13 |
| *g149* | ttttattttatcttttatttaaagaattataaaatttgagaaacaaaactA | 124037 | - | 32 | Primary | 24 | >100 | 0,26 |
| *g154* | tttacatataatacttagttatttttaagatattaaaaaacataaaaagtG | 127733 | + | 72 | Secondary | 56 | >100 | 0,91 |
| *g198* | gggaagaatttaatttataaattttttaaaaaattattaactaaaagaatA | 163697 | - | 15 | Primary | 29 | >100 | 1,58 |
| *g202* | attaccttaaataattgtagttatgatataactattttaaactaattcttA | 165686 | + | 50 | Primary | NA | NA | NA |
| *g213* | cttataatatatatttgaaaaatgagttttcttttacttagaacaattaaA | 181670 | - | 29 | Primary | 17 | 18 | 0,98 |
| *g240* | tttaaatctctagatttgttacaaaaataatctactaaaaacattagataT | 206330 | + | NA | Antisense | 15 | 4,75 | 1,61 |
| *g242* | gtaatttttaaaataaaaaagagaaataaattttttaaaacttaatatttA | 207906 | - | NA | Antisense | 15 | 2,88 | 0,89 |
| *g246* | aaacaatattaattttcaaattaaaaactaaaataagtaaactagatattA | 209515 | + | 44 | Primary | 698,5 | >100 | 0,84 |
| *g273* | gaacaaacagatgttattcttaacttattagaagatattgaaacattcttA | 229263 | - | NA | Internal | 3646 | >100 | 0,63 |
| *g002* | tataaatattaattctcattaaaataaaagaaatcaactaataaataattA | 353 | + | 56 | Primary | 128,5 | >100 | 0,85 |
| *g083* | aattgcatttaattgtcattaccttaaatgaaatataagaatatattattA | 68271 | - | 30 | Primary | NA | NA | NA |
| *g119* | ttttttcttttttgagtttcaagatgaagttttctggttaaaataaaaacA | 93887 | - | 38 | Primary | NA | NA | NA |
| *g226* | aatctcaaaaaagagttgcaaagaaagagaaaaatagttataatggagaaG | 196022 | - | 196 | Primary | NA | NA | NA |
| *g233* | ttattatttctttattctttaaattttaaaagataatcaaaaaattcatcA | 201544 | - | NA | Antisense | 47 | 24,5 | 0,91 |
| *g234* | gaattttttgattatcttttaaaatttaaagaataaagaaataataaattA | 201598 | + | 151 | Secondary | 102 | 15,57 | 2,16 |
| *g251* | gttatctactaatactctggctttcaagagttttgacatatctaatttttG | 213250 | - | NA | Antisense | 20 | >100 | 0,69 |
