# Supplementary material for: Transcription Profiling of Bacillus subtilis Cells Infected with AR9, a Giant Phage Encoding Two Multisubunit RNA Polymerases
Source: mBio. 2017 Feb 14;8(1):e02041-16. doi: 10.1128/mBio.02041-16 (PMC5312081; doi:10.1128/mBio.02041-16)
Supplement: TABLE S4 [file mbo001173180st4.docx]

**Table S4. Transcription classes of AR9 genes.**

| **Gene** | **Protein** | **5' FPKM** | **20' FPKM** | **40' FPKM** |
| --- | --- | --- | --- | --- |
| ***g001*** | hypothetical protein | 67,35 | 274,13 | 558,72 |
| ***g002*** | metallophosphatase | 30,88 | 148,29 | 254,06 |
| ***g003*** | hypothetical protein | 61,41 | 562 | 551,54 |
| ***g004*** | DNA processing chain A dprA protein | 47,84 | 468,48 | 555,6 |
| ***g005*** | hypothetical protein | 67,19 | 409,21 | 424,37 |
| ***g006*** | hypothetical protein | 42,74 | 271,75 | 326,04 |
| ***g007*** | DnaB-like replicative helicase | 15,01 | 110,3 | 241,55 |
| ***g008*** | hypothetical protein | 103,57 | 1543,72 | 1339,6 |
| ***g009*** | hypothetical protein | 135,42 | 1031,48 | 828,48 |
| ***g010*** | base plate wedge protein | 0,94 | 13,35 | 203,65 |
| ***g011*** | hypothetical protein | 0,66 | 14,87 | 329,71 |
| ***g012*** | tail fiber protein | 8,37 | 16,92 | 158,6 |
| ***g013*** | zincin-like protease | 50,09 | 174,37 | 252,87 |
| ***g014*** | DNA binding protein | 108,97 | 788,51 | 1141,50 |
| ***g015*** | hypothetical protein | 32,43 | 318,8 | 266,47 |
| ***g016*** | RecD/TraA family helicase | 45,52 | 271,7 | 338,95 |
| ***g017*** | metallophosphatase | 34,85 | 187,98 | 413,23 |
| ***g018*** | hypothetical protein | 50,9 | 541,63 | 744,89 |
| ***g019*** | hypothetical protein | 226,44 | 2366,13 | 2044,87 |
| ***g020*** | N-acetylmuramyl-L-alanine amidase | 1,65 | 226,07 | 3848,41 |
| ***g021*** | hypothetical protein | 13,41 | 1416,23 | 3469,23 |
| ***g022*** | metallo-beta-lactamase superfamily protein | 2,35 | 153,55 | 1091,22 |
| ***g023*** | hypothetical protein | 3,38 | 3883,28 | 16149,9 |
| ***g024*** | DNA gyrase subunit B | 34,13 | 232,91 | 394,4 |
| ***g025*** | mannosyl-glycoendo-beta-N-acetylglucosaminidase | 83,68 | 255,43 | 217,15 |
| ***g026*** | hypothetical protein | 81,11 | 647,32 | 376,09 |
| ***g027*** | DNA binding protein | 28,31 | 140,08 | 184,75 |
| ***g028*** | AAA family ATPase | 88,02 | 463,56 | 378,46 |
| ***g029*** | zincin-like protease fused to vWA domain | 77,25 | 594,93 | 465 |
| ***g030*** | hypothetical protein | 24,53 | 102,66 | 86,87 |
| ***g031*** | hypothetical protein | 79,58 | 146,93 | 151,97 |
| ***g032*** | HNH nuclease | 13,93 | 39,54 | 214,99 |
| ***g033*** | hypothetical protein | 160,93 | 1363,97 | 474,27 |
| ***g034*** | hypothetical protein | 29,04 | 143,53 | 121,38 |
| ***g035*** | hypothetical protein | 25,63 | 130,78 | 142,38 |
| ***g036*** | hypothetical protein | 40,69 | 251,79 | 141,56 |
| ***g037*** | hypothetical protein | 49,14 | 147,07 | 205,97 |
| ***g038*** | hypothetical protein | 57,08 | 210,63 | 255,32 |
| ***g039*** | hypothetical protein | 2443,96 | 3683,43 | 2614,81 |
| ***g040*** | hypothetical protein | 4545,87 | 13401,6 | 4926,09 |
| ***g041*** | Zn finger protein of DnaJ family | 111,46 | 181,49 | 172,04 |
| ***g042*** | Zn finger protein of DnaJ family | 46,23 | 109,03 | 130,23 |
| ***g043*** | hypothetical protein | 26,61 | 289,23 | 176,80 |
| ***g044*** | DNA gyrase subunit A | 81,72 | 393,73 | 356,93 |
| ***g045*** | hypothetical protein | 44,26 | 391,65 | 229,07 |
| ***g046*** | hypothetical protein | 150,75 | 774,05 | 1036,65 |
| ***g047*** | hypothetical membrane protein | 684,4 | 1034,78 | 944,24 |
| ***g048*** | hypothetical membrane protein | 7458,91 | 6577,2 | 4570,08 |
| ***g049*** | peptidoglycan-binding protein | 722,44 | 971,96 | 662 |
| ***g050*** | hypothetical protein | 3910,28 | 4692,08 | 4906,57 |
| ***g051*** | Zincin protease, SprT-like family | 1,59 | 44,9 | 1222,74 |
| ***g052*** | hypothetical protein | 0,26 | 9,64 | 210,7 |
| ***g053*** | hypothetical protein | 3,44 | 24,15 | 1970,8 |
| ***g054*** | hypothetical protein | 0 | 11,14 | 349,53 |
| ***g055*** | hypothetical protein | 0,5 | 7,37 | 370,92 |
| ***g056*** | hypothetical protein | 0,5 | 12,96 | 1061,8 |
| ***g057*** | virion protein | 0,58 | 46,42 | 1619,97 |
| ***g058*** | virion protein | 0,1 | 14,73 | 487,47 |
| ***g059*** | virion protein | 0 | 85,88 | 1363,33 |
| ***g060*** | virion protein | 0,61 | 18,17 | 759,77 |
| ***g061*** | virion protein | 3,17 | 199,99 | 4637,08 |
| ***g062*** | hypothetical protein | 0,6 | 5,07 | 156,44 |
| ***g063*** | Zn finger protein | 769,62 | 2417,57 | 3384,13 |
| ***g064*** | hypothetical protein | 100,33 | 282,39 | 751,69 |
| ***g065*** | hypothetical protein | 66,69 | 267,63 | 798,76 |
| ***g066*** | hypothetical protein | 88,58 | 552,31 | 2070,65 |
| ***g067*** | poly-gamma-glutamate hydrolase | 18,97 | 122,73 | 669,24 |
| ***g068*** | von Willebrand factor type A domain containing protein | 0,96 | 105,57 | 431,49 |
| ***g069*** | hypothetical protein | 248,89 | 305,56 | 258,23 |
| ***g070*** | hypothetical protein | 827,06 | 890,66 | 286,31 |
| ***g071*** | hypothetical protein | 3532,11 | 7659,42 | 9728,56 |
| ***g072*** | hypothetical protein | 796,94 | 887,97 | 1720,68 |
| ***g073*** | hypothetical protein | 1123,01 | 3745,7 | 1861,79 |
| ***g074*** | NUDIX hydrolase | 265,55 | 686,86 | 825,68 |
| ***g075*** | PhoH protein | 1,3 | 60,42 | 610,93 |
| ***g076*** | hypothetical protein | 2,09 | 52,16 | 1916,26 |
| ***g077*** | virion protein | 2,2 | 141,73 | 7677,9 |
| ***g078*** | HD family phosphohydrolase domain fused to DNA-directed RNA polymerase beta subunit, C-terminus | 0,86 | 39,67 | 590,06 |
| ***g079*** | homing endonuclease | 0,4 | 24,46 | 407,33 |
| ***g080*** | hypothetical protein | 2,13 | 81,43 | 1442,77 |
| ***g081*** | hypothetical membrane protein | 0,98 | 8,27 | 627,88 |
| ***g082*** | putative holin | 2,33 | 26,33 | 2700,72 |
| ***g083*** | AAA family ATPase | 11,52 | 85,75 | 282,23 |
| ***g084*** | virion protein | 29,32 | 389,94 | 1015,42 |
| ***g085*** | Zn finger protein | 195 | 2779,09 | 3225,67 |
| ***g086*** | UvsX-like recombinase | 10,81 | 496,77 | 920,29 |
| ***g087*** | hypothetical protein | 61,13 | 294,76 | 296,9 |
| ***g088*** | hypothetical protein | 23,31 | 361,03 | 318 |
| ***g089*** | DNA-directed RNA polymerase beta subunit, C-terminus | 32,24 | 227,46 | 398,23 |
| ***g090*** | hypothetical protein | 699,28 | 5465,66 | 9026,42 |
| ***g091*** | hypothetical protein | 52,59 | 165,32 | 252,3 |
| ***g092*** | hypothetical protein | 120,93 | 600,81 | 692,96 |
| ***g093*** | DNA binding protein | 940,69 | 5696,25 | 5221,21 |
| ***g094*** | hypothetical protein | 311,47 | 1502,41 | 1047,48 |
| ***g095*** | NAD-dependent DNA-ligase | 32,66 | 300,84 | 662,44 |
| ***g096*** | virion protein | 0 | 19,53 | 275,24 |
| ***g097*** | hypothetical protein | 2581,88 | 2612,93 | 5891,08 |
| ***g098*** | hypothetical protein | 1626,2 | 1440,99 | 9234,79 |
| ***g099*** | non-homologous end joining protein | 221,48 | 1116,31 | 878,65 |
| ***g100*** | hypothetical protein | 207,73 | 516,86 | 335,12 |
| ***g101*** | hypothetical protein | 1342,01 | 2236,42 | 2206,77 |
| ***g102*** | hypothetical protein | 153,87 | 431,06 | 168,86 |
| ***g103*** | DNA binding protein HU | 26,85 | 7522,03 | 52104,6 |
| ***g104*** | uracil-DNA-glycosylase inhibitor | 422,29 | 681,52 | 2065,59 |
| ***g105*** | DNA-directed RNA polymerase beta subunit, N-terminus | 21,55 | 122,09 | 202,16 |
| ***g106*** | hypothetical protein | 691,23 | 4063,52 | 5471,2 |
| ***g107*** | hypothetical protein | 20,06 | 180,8 | 384,35 |
| ***g108*** | hypothetical protein | 280,08 | 983,59 | 687,29 |
| ***g109*** | hypothetical membrane protein | 947,62 | 851,92 | 539,1 |
| ***g110*** | N-glycosylase, DNA lyase | 1903,25 | 4562,53 | 3248,78 |
| ***g111*** | hypothetical protein | 4,88 | 1,9 | 0 |
| ***g112*** | virion protein | 6,97 | 41,5 | 1401,74 |
| ***g113*** | hypothetical protein | 0 | 32,14 | 1588,17 |
| ***g114*** | prohead core scaffold protein and protease | 0,26 | 4,14 | 114,3 |
| ***g115*** | virion protein | 0,95 | 10,35 | 796,42 |
| ***g116*** | hypothetical protein | 1,67 | 61,01 | 1331,16 |
| ***g117*** | precursor of major head subunit | 1,4 | 53,65 | 3598,58 |
| ***g118*** | zincin protease | 1,27 | 23,64 | 1422,53 |
| ***g119*** | hypothetical protein | 76,9 | 329,61 | 10726,5 |
| ***g120*** | hypothetical protein | 313,13 | 236,97 | 167,24 |
| ***g121*** | terminase large subunit | 88,28 | 177,26 | 1929,77 |
| ***g122*** | hypothetical protein | 0,98 | 25,75 | 196,05 |
| ***g123*** | hypothetical protein | 0,43 | 141,2 | 456,08 |
| ***g124*** | hypothetical protein | 1,44 | 498,61 | 1682,87 |
| ***g125*** | DNA-directed RNA polymerase beta'-subunit, C-terminus | 0,9 | 35,3 | 1964,38 |
| ***g126*** | contractile tail sheath structural protein | 1,24 | 27,99 | 2915,72 |
| ***g127*** | virion protein | 5,33 | 176,15 | 3562,4 |
| ***g128*** | portal vertex protein | 0,25 | 113,69 | 944,46 |
| ***g129*** | hypothetical protein | 20,88 | 127,97 | 318,87 |
| ***g130*** | hypothetical secreted/membrane protein | 288,73 | 1273,9 | 4361,18 |
| ***g131*** | DnaQ-like 3'-5' exonuclease | 29,9 | 222,37 | 571,58 |
| ***g132*** | DNA polymerase with DnaQ-like 3'-5' exonuclease and partial catalytic domain | 14,39 | 121,84 | 292,02 |
| ***g133*** | hypothetical protein | 213,01 | 2630,93 | 7440,03 |
| ***g134*** | hypothetical protein | 198,18 | 1346,72 | 3178,96 |
| ***g135*** | hypothetical protein | 100,9 | 701,91 | 1270,19 |
| ***g136*** | Zn finger protein | 56,24 | 337,25 | 1299,72 |
| ***g137*** | hypothetical protein | 47,14 | 364,18 | 872,07 |
| ***g138*** | hypothetical secreted/membrane protein | 17,13 | 318,26 | 365,49 |
| ***g139*** | SbcC ATPase | 9,95 | 88,57 | 183,12 |
| ***g140*** | YbiA family NAD-utilizing enzyme | 26,81 | 197,65 | 457,3 |
| ***g141*** | Zn finger protein | 310,33 | 1395,33 | 3408,11 |
| ***g142*** | hypothetical protein | 0,76 | 165,64 | 7042,17 |
| ***g143*** | hypothetical protein | 38,95 | 531,28 | 1798,53 |
| ***g144*** | hypothetical protein | 0,43 | 42,13 | 245,74 |
| ***g145*** | DNA-directed RNA polymerase beta'-subunit, N-terminus | 0 | 38,29 | 298,47 |
| ***g146*** | Hef-like homing endonuclease | 0,35 | 43,11 | 721,51 |
| ***g147*** | homing endonuclease | 0,54 | 47,65 | 469,36 |
| ***g148*** | hypothetical protein | 611,44 | 1953,49 | 5530,79 |
| ***g149*** | DNA binding protein | 28,13 | 174,66 | 453,2 |
| ***g150*** | hypothetical membrane protein | 79,91 | 271,35 | 986,83 |
| ***g151*** | 5'(3')-deoxyribonucleotidase | 172,11 | 787,9 | 2349,19 |
| ***g152*** | DNA polymerase catalytic subunit | 16,61 | 103,98 | 257,43 |
| ***g153*** | virion protein | 7,13 | 2010,41 | 66117,3 |
| ***g154*** | DNA-directed RNA polymerase beta' subunit, C-terminus; | 33,24 | 144,73 | 441,9 |
| ***g155*** | hypothetical protein | 65,29 | 250,01 | 454,7 |
| ***g156*** | DNA/RNA helicase of superfamily II | 42,91 | 220,78 | 510,91 |
| ***g157*** | oligoribonuclease NrnB | 46,5 | 262,23 | 489,3 |
| ***g158*** | 3'-5' exonuclease | 35,66 | 273,1 | 293,03 |
| ***g159*** | hypothetical protein | 1344,00 | 16206,40 | 13982,10 |
| ***g160*** | hypothetical protein | 479,6 | 565,83 | 3793,39 |
| ***g161*** | hypothetical protein | 114,74 | 644,36 | 2750,28 |
| ***g162*** | hypothetical protein | 1592,06 | 11944,8 | 17250,7 |
| ***g163*** | hypothetical protein | 0 | 303,26 | 5367,03 |
| ***g164*** | hypothetical protein | 0,61 | 178,14 | 3508,17 |
| ***g165*** | virion protein | 0 | 19,44 | 371,19 |
| ***g166*** | hypothetical protein | 0 | 15,04 | 353,59 |
| ***g167*** | hypothetical protein | 0 | 380,91 | 6450,52 |
| ***g168*** | hypothetical protein | 7,49 | 1209,96 | 4120,92 |
| ***g169*** | hypothetical protein | 258,46 | 166,32 | 136,21 |
| ***g170*** | hypothetical protein | 120,05 | 366,65 | 633,19 |
| ***g171*** | hypothetical protein | 128,78 | 694,19 | 564,26 |
| ***g172*** | Zn finger protein | 79,88 | 814,38 | 594,04 |
| ***g173*** | hypothetical membrane protein | 500,68 | 1624,30 | 2928,12 |
| ***g174*** | AAA family ATPase | 119,59 | 348,97 | 432,27 |
| ***g175*** | hypothetical protein | 117,16 | 2114,37 | 1370,53 |
| ***g176*** | hypothetical protein | 41,17 | 273,28 | 996,08 |
| ***g177*** | hypothetical protein | 28,31 | 355,57 | 2214,74 |
| ***g178*** | hypothetical protein | 81,51 | 837,88 | 906,07 |
| ***g179*** | hypothetical membrane protein | 139,19 | 1173,51 | 2618,58 |
| ***g180*** | hypothetical sereted/membrane protein | 99,22 | 6068,60 | 6560,55 |
| ***g181*** | stomatin/prohibitin superfamily protein | 31,12 | 603,43 | 1651,15 |
| ***g182*** | hypothetical protein | 131,32 | 319,82 | 960,34 |
| ***g183*** | Zn finger protein | 52,87 | 367,43 | 409,75 |
| ***g184*** | hypothetical protein | 71,19 | 484,59 | 557,53 |
| ***g185*** | hypothetical protein | 156,09 | 716,39 | 1260,77 |
| ***g186*** | hypothetical protein | 52,84 | 260,72 | 301,28 |
| ***g187*** | hypothetical protein | 716,15 | 3962,74 | 4283,58 |
| ***g188*** | Hef-like homing endonuclease | 2,74 | 99,4 | 400,28 |
| ***g189*** | DNA-directed RNA polymerase beta subunit, N-terminus | 0 | 24 | 390 |
| ***g190*** | HNH homing endonuclease with DNA-binding AP2 domain | 290,69 | 405,88 | 426,91 |
| ***g191*** | hypothetical protein | 0 | 54,54 | 893,28 |
| ***g192*** | hypothetical protein | 0 | 7,01 | 97,21 |
| ***g193*** | virion protein | 0,63 | 30,03 | 828,98 |
| ***g194*** | hypothetical protein | 0 | 49,48 | 1507,59 |
| ***g195*** | N-acetylmuramoyl-L-alanine amidase | 0 | 19,84 | 953,91 |
| ***g196*** | hypothetical protein | 0,62 | 10,87 | 432,8 |
| ***g197*** | virion protein | 0,72 | 22,8 | 680,34 |
| ***g198*** | virion protein | 0 | 16,07 | 235,55 |
| ***g199*** | hypothetical protein | 0 | 1,24 | 219,92 |
| ***g200*** | hypothetical protein | 1250,32 | 5707,8 | 8701,41 |
| ***g201*** | hypothetical protein | 26,37 | 225,45 | 177,57 |
| ***g202*** | hypothetical protein | 87,52 | 193,16 | 480,53 |
| ***g203*** | virion protein | 409,18 | 1267,07 | 1821,61 |
| ***g204*** | virion protein | 1,36 | 21,57 | 309,91 |
| ***g205*** | virion protein | 1,61 | 236,78 | 1425,47 |
| ***g206*** | hypothetical protein | 0 | 502,3 | 3409,73 |
| ***g207*** | hypothetical protein | 0 | 797,45 | 5480,74 |
| ***g208*** | Holliday junction resolvase RusA | 0,61 | 146,81 | 777,12 |
| ***g209*** | hypothetical protein | 0,8 | 197 | 626,63 |
| ***g210*** | predicted P-loop kinase/ATPase | 1,12 | 1124,09 | 4404,3 |
| ***g211*** | hypothetical protein | 0,39 | 39,08 | 889,48 |
| ***g212*** | hypothetical protein | 0,49 | 10,48 | 159,65 |
| ***g213*** | virion protein | 2,01 | 23,21 | 200,71 |
| ***g214*** | minor virion structural protein | 0,69 | 20,67 | 1426,72 |
| ***g215*** | virion protein | 0,29 | 1,6 | 2,69 |
| ***g216*** | virion protein | 0,53 | 84,63 | 4748,49 |
| ***g217*** | hypothetical protein | 1,06 | 27,16 | 485,96 |
| ***g218*** | hypothetical protein | 0,49 | 57,94 | 947,53 |
| ***g219*** | hypothetical secreted/membrane protein | 95,35 | 1529,24 | 1403,61 |
| ***g220*** | hypothetical protein | 33,08 | 125,47 | 567,98 |
| ***g221*** | dCMP deaminase | 17,8 | 112,73 | 126,31 |
| ***g222*** | ribonucleotide-diphosphate reductase beta subunit NrdF | 5,77 | 100,72 | 725,48 |
| ***g223*** | ribonucleotide-diphosphate reductase alpha subunit NrdE | 7,88 | 143,39 | 620,71 |
| ***g224*** | flavodoxin co-factor of ribonucleotide-diphosphate reductase, NrdI | 21,23 | 326,52 | 1040,59 |
| ***g225*** | DNA primase | 10,83 | 85,29 | 462,22 |
| ***g226*** | DNA-directed RNA polymerase subunit | 36,52 | 146,77 | 611,16 |
| ***g227*** | hypothetical protein | 0 | 32,12 | 182,48 |
| ***g228*** | chaperonin GroEL | 0,49 | 41,95 | 621,1 |
| ***g229*** | hypothetical protein | 0 | 153,55 | 1157,05 |
| ***g230*** | hypothetical protein | 0 | 122,41 | 576,02 |
| ***g231*** | hypothetical protein | 1,23 | 87,2 | 485,68 |
| ***g232*** | base plate wedge subunit | 0 | 59,33 | 358,95 |
| ***g233*** | endonuclease fused to N-terminal Zn-finger domain | 0,47 | 141,28 | 1092,1 |
| ***g234*** | hypothetical protein | 295,8 | 155,76 | 83,77 |
| ***g235*** | hypothetical protein | 471,55 | 364,23 | 174,11 |
| ***g236*** | hypothetical protein | 421,72 | 570,15 | 351,45 |
| ***g237*** | hypothetical protein | 128,96 | 2209,97 | 18724,4 |
| ***g238*** | guanylate kinase | 237,16 | 1193,39 | 3150,79 |
| ***g239*** | hypothetical protein | 60,68 | 308,83 | 1533,01 |
| ***g240*** | hypothetical secreted/membrane protein | 1,92 | 303,68 | 3028,26 |
| ***g241*** | ribonuclease H | 323,45 | 725,32 | 1580,06 |
| ***g242*** | hypothetical protein | 0 | 55,28 | 751,86 |
| ***g243*** | hypothetical protein | 1,53 | 572,32 | 2326,32 |
| ***tRNA*** | asparagine tRNA | 0,00 | 204422,00 | 242943,00 |
| ***g244*** | hypothetical secreted/membrane protein | 0 | 2595,93 | 16225,2 |
| ***g245*** | hypothetical protein | 1,79 | 812,45 | 3153,87 |
| ***g246*** | hypothetical protein | 0,43 | 96,43 | 272,35 |
| ***g247*** | hypothetical secreted/membrane protein | 0 | 69,66 | 1855,52 |
| ***g248*** | hypothetical membrane protein | 305,11 | 466,77 | 1243,31 |
| ***g249*** | DNA binding protein with Zn finger domain | 267,31 | 385,33 | 780,16 |
| ***g250*** | hypothetical protein | 384,99 | 814,69 | 1338,02 |
| ***g251*** | virion protein | 0,82 | 90,36 | 1246,15 |
| ***g252*** | hypothetical protein | 10,9 | 95,6 | 2028,09 |
| ***g253*** | hypothetical protein | 486,64 | 413,75 | 1591,98 |
| ***g254*** | hypothetical protein | 798,5 | 782,38 | 968,56 |
| ***g255*** | hypothetical protein | 296,73 | 483,4 | 468,89 |
| ***g256*** | hypothetical protein | 330,44 | 692,16 | 418,92 |
| ***g257*** | DNA binding protein | 1389,57 | 2556,05 | 1263,29 |
| ***g258*** | hypothetical secreted/membrane protein | 3408,06 | 8532 | 2568,82 |
| ***g259*** | virion protein | 1,38 | 103,64 | 1338,2 |
| ***g260*** | virion protein | 0,49 | 33,95 | 701,62 |
| ***g261*** | hypothetical protein | 4,21 | 32,42 | 745,57 |
| ***g262*** | hypothetical protein | 835,24 | 2934,05 | 1826,01 |
| ***g263*** | hypothetical protein | 964,04 | 1860,01 | 1625,57 |
| ***g264*** | DNA-directed RNA polymerase beta’ subunit, middle-terminus | 1,71 | 52,52 | 595,98 |
| ***g265*** | Hef-like homing endonuclease | 13,58 | 79,77 | 298,04 |
| ***g266*** | hypothetical protein | 724,88 | 885,17 | 2977,61 |
| ***g267*** | hypothetical protein | 427,85 | 883,62 | 2339,63 |
| ***g268*** | hypothetical protein | 159,5 | 462,89 | 830,17 |
| ***g269*** | hypothetical protein | 99,85 | 504,49 | 581,77 |
| ***g270*** | DNA-directed RNA polymerase beta' subunit, N-terminus | 19,51 | 109,7 | 271,41 |
| ***g271*** | HNH homing endonuclease | 26,76 | 329,88 | 366,63 |
| ***g272*** | N-acetylmuramoyl-L-alanin Amidase with peptidoglycan binding domains | 0,97 | 41,04 | 259,12 |
| ***g273*** | virion protein | 1,44 | 153,92 | 225,27 |
| ***g274*** | hypothetical protein | 0 | 0 | 309,18 |
| ***g275*** | virion protein, contains PKD-like repeats | 0,42 | 5,82 | 165,04 |
| ***g276*** | virion protein , contains Ig-like domain | 1,38 | 28,65 | 541,47 |
| ***g277*** | virion protein | 0 | 39,44 | 725,86 |
| ***g278*** | virion protein | 0,47 | 5,66 | 635,09 |
| ***g279*** | virion protein | 0,23 | 4,16 | 149,38 |
| ***g280*** | hypothetical protein | 0,21 | 12,91 | 330,42 |
| ***g281*** | virion protein | 0 | 23,47 | 762,73 |
| ***g282*** | portal protein | 0 | 15,06 | 330,8 |
| ***g283*** | hypothetical protein | 116547,06 | 1018650,00 | 283868,00 |
| ***g284*** | hypothetical protein | 152,95 | 326,61 | 130,88 |
| ***g285*** | hypothetical protein | 716,71 | 1273,62 | 658,24 |
| ***g286*** | hypothetical protein | 655,22 | 1422 | 633,18 |
| ***g287*** | hypothetical protein | 277,92 | 446,88 | 342,03 |
| ***g288*** | hypothetical protein | 2237,13 | 3614,25 | 1353,65 |
| ***g289*** | hypothetical protein | 3941,03 | 2256,92 | 1117,44 |
| ***g290*** | hypothetical protein | 1192,78 | 2146,04 | 405,72 |
| ***g291*** | hypothetical protein | 6486,49 | 8110,03 | 2033,13 |
| ***g292*** | hypothetical membrane protein | 775,66 | 238,64 | 210,34 |
