# Supplementary material for: Transcription Profiling of Bacillus subtilis Cells Infected with AR9, a Giant Phage Encoding Two Multisubunit RNA Polymerases
Source: mBio. 2017 Feb 14;8(1):e02041-16. doi: 10.1128/mBio.02041-16 (PMC5312081; doi:10.1128/mBio.02041-16)
Supplement: TABLE S5 [file mbo001173180st5.docx]

**Table S5. List of *B. subtilis* 168 genes with significantly changed expression levels during AR9 infection.**

| gene | FPKM values | | | protein | type | KEGG | regulated by |
| --- | --- | --- | --- | --- | --- | --- | --- |
|  | **5'** | **20'** | **40'** |  |  |  |  |
| *glpT* | 58,82 | 96,44 | 1333,91 | glycerol-3-phosphate transporter | transporter | bsu02010 | σ^A^, GlpP activator |
| *lmrA* | 0 | 16,25 | 33,93 | TetR family transcriptional regulator | regulator |  | σ^A^, LmrA repressor |
| *ydaD* | 0 | 6,44 | 15,06 | general stress protein 39 | factor |  | σ^B^ |
| *aseR* | 0 | 29,06 | 59,16 | ArsR family transcriptional regulator | regulator |  |  |
| *gmuC* | 0 | 6,03 | 4,41 | PTS system-oligo-beta-mannoside-specific transporter subunit IIC | transporter | bsu02060 | σ^A^, CcpA repressor |
| *pspA* | 2686,96 | 361,81 | 128,57 | phage shock protein A homolog | factor |  | σ^W^ |
| *ydjM* | 72,14 | 1179,17 | 1013,88 | hypothetical protein | - |  |  |
| *ydjN* | 7,31 | 176,81 | 143,91 | hypothetical protein | membrane  component |  |  |
| *pbuG* | 10,42 | 12,37 | 121,81 | guanine/hypoxanthine permease PbuG | transporter |  | σ^A^, PurR repressor |
| *yfiN* | 0 | 5,62 | 2,60 | transport permease YfiN | transporter |  |  |
| *sat* | 0 | 0,88 | 12,18 | sulfate adenylyltransferase | enzyme | bsu01040 bsu00261 bsu00270 bsu00311 | σ^A^ |
| *sspD* | 219,10 | 120,37 | 0 | small acid-soluble spore protein D | factor |  | σ^G^ |
| *misc_RNA_*  *29* | 184,62 | 1673,62 | 0 | RNA motif | RNA motif |  | σ^A^ |
| *ynzK* | 0 | 3,61 | 35,65 | hypothetical protein | membrane  component |  |  |
| *yobH* | 0 | 13,75 | 5,48 | DNA repair protein | enzyme |  | σ^A^, 2 LexA repressor |
| *yocH* | 50,81 | 356,77 | 2168,71 | cell wall-binding protein YocH | factor | bsu00121 bsu01053bbsu01110 bsu01130 | σ^A^, WalR activator |
| *ypzG* | 0 | 585,77 | 345,78 | hypothetical protein | - |  |  |
| *ypuD* | 1667,28 | 323,97 | 136,93 | hypothetical protein | - |  | σ^A^, LexA repressor |
| *yqkK* | 17,48 | 129,63 | 0 | hypothetical protein | regulator |  |  |
| *aspA* | 19,05 | 59,58 | 151,38 | aspartate ammonia-  lyase | enzyme | bsu00250  bsu00260  bsu00270  bsu02030 |  |
| *pstBB* | 0 | 20,44 | 11,74 | phosphate import ATP-binding protein PstB 1 | transporter | bsu02010 | σ^A^, PhoR repressor, PhoP activator |
| *pstS* | 0 | 12,91 | 19,46 | phosphate-binding protein PstS | transporter | bsu02010 bsu02020 |  |
| *arsR* | 0 | 49,79 | 25,99 | arsenical resistance operon repressor | regulator | bsu03018 | - |
| *yqbK* | 0 | 6,37 | 3,11 | hypothetical protein | - |  |  |
| *yraB* | 0 | 3,82 | 25,16 | MerR family transcriptional regulator | regulator |  |  |
| *misc_RNA_*  *45* | 1856,84 | 2856,02 | 312,58 | T-box | RNA motif |  | σ^A^ |
| *bioB* | 166,14 | 41,42 | 13,85 | biotin synthase | enzyme | bsu00780 | σ^A^ |
| *trnB-Val* | 0 | 2632,08 | 4809,17 | trnB-Val | tRNA |  | 2 σ^A^ |
| *rbsC* | 23,28 | 33,20 | 209,47 | ribose transport system permease RbsC | transporter | bsu02010 | σ^A^, CcpA repressor, AbrB activator |
| *ywnC* | 59,98 | 67,97 | 663,83 | hypothetical protein | membrane  component |  |  |
| *misc_RNA_*  *57* | 400,58 | 1650,55 | 2779,34 | T-box | RNA motif |  |  |
| *misc_RNA_*  *60* | 1823,83 | 333,9 | 0 | RNA motif | RNA motif |  |  |
| *yxjI* | 603,62 | 163,19 | 60,35 | hypothetical protein | regulator |  | σ^A^, DegU repressor |
| *yxjA* | 50,55 | 30,53 | 334,05 | purine nucleoside transport protein | transporter |  |  |
